# Transgelin-2, a novel cancer stem cell-related biomarker, is a diagnostic and therapeutic target for biliary tract cancer

**DOI:** 10.1186/s12885-024-12082-3

**Published:** 2024-03-20

**Authors:** Jung Hyun Jo, Soo Been Park, Joowon Chung, Taeyun Oh, Hee Seung Lee, Moon Jae Chung, Jeong Youp Park, Seungmin Bang, Seung Woo Park, Dawoon E. Jung, Si Young Song

**Affiliations:** 1 Division of Gastroenterology, Department of Internal Medicine, Yonsei University College of Medicine, Seoul, Korea; 2 Department of Internal Medicine, Nowon Eulji Medical Center, Eulji University School of Medicine, Seoul, Korea; 3 Cowell Biodigm Co., Ltd., Seoul, Korea; 4 Institute of Gastroenterology, Yonsei University College of Medicine, Seoul, Korea

**Keywords:** Biliary tract cancer, Transgelin-2, Cancer stem cell, Therapy-resistance, Cancer-associated fibroblasts

## Abstract

**Background:**

Biliary tract cancer (BTC) is a relatively rare but aggressive gastrointestinal cancer with a high mortality rate. Cancer stem cell (CSC) populations play crucial roles in tumor biology and are responsible for the low response to anti-cancer treatment and the high recurrence rate. This study investigated the role of Transgelin-2 (*TAGLN2*), overexpressed in CSC in BTC cells, and analyzed its expression in patient tissues and serum to identify potential new targets for BTC.

**Methods:**

*TAGLN2* expression was suppressed by small-interfering or short hairpin RNAs, and its effects on tumor biology were assessed in several BTC cell lines. Furthermore, the effects of *TAGLN2* silencing on gemcitabine-resistant BTC cells, differentially expressed genes, proteins, and sensitivity to therapeutics or radiation were assessed. TAGLN2 expression was also assessed using western blotting and immunohistochemistry in samples obtained from patients with BTC to validate its clinical application.

**Results:**

Suppression of *TAGLN2* in BTC cell lines decreased cell proliferation, migration, invasion, and tumor size, in addition to a reduction in CSC features, including clonogenicity, radioresistance, and chemoresistance. TAGLN2 was highly expressed in BTC tissues, especially in cancer-associated fibroblasts in the stroma. Patients with a low stromal immunohistochemical index had prolonged disease-free survival compared to those with a high stromal immunohistochemical index (11.5 vs. 7.4 months, *P* = 0.013). TAGLN2 expression was higher in the plasma of patients with BTC than that in those with benign diseases. TAGLN2 had a higher area under the curve (0.901) than CA19-9, a validated tumor biomarker (0.799; *P* < 0.001).

**Conclusion:**

TAGLN2 plays a critical role in promoting BTC cell growth and motility and is involved in regulating BTC stemness. Silencing *TAGLN2* expression enhanced cell sensitivity to radiation and chemotherapeutic drugs. The expression of TAGLN2 in patient tissue and plasma suggests its potential to serve as a secretory biomarker for BTC. Overall, targeting *TAGLN2* could be an appropriate therapeutic strategy against advanced cancer following chemotherapy failure.

**Supplementary Information:**

The online version contains supplementary material available at 10.1186/s12885-024-12082-3.

## Background

Biliary tract cancer (BTC) is a relatively rare but aggressive gastrointestinal cancer with a high mortality rate [1]. Surgical resection is the only curative modality for BTC [2]; although complete resection has been achieved, the recurrence of BTC is high in patients with resected BTC [3] with a poor 5-year survival of 20–30% [4, 5]. Furthermore, most tumors metastasize at diagnosis, imposing difficulties in standard treatments [6]. Therefore, palliative treatments other than systemic chemotherapy or radiotherapy are the only option for patients with unresectable or metastatic BTC. The combined use of gemcitabine and cisplatin is recommended as first-line treatment for these patients [7]. This combination chemotherapy improves progression-free and overall survival, but the median overall survival is no longer than one year in metastatic BTC [8].

Cancer stem cells (CSCs) play crucial roles in tumor initiation, progression, and relapse. Most CSCs show characteristics of epithelial-mesenchymal transition (EMT); therefore, CSCs in BTCs are highly desmoplastic, morphologically heterogenic, aggressive, and resistant to chemotherapy. Previous studies have demonstrated that CSCs in BTC are responsible for the low response to anti-cancer treatment and high recurrence rate [9]. Identifying specific CSC markers for BTC is a dynamic field of research. Several potential markers have been proposed, including CD24, CD44, EpCAM, CD133, and aldehyde dehydrogenase 1 [10–13]. However, the phenotypic heterogeneity and cellular plasticity of CSCs hinder their application [14]. Therefore, identifying a representative CSC marker for BTC and understanding the characteristics of CSCs (especially EMT) in BTC are crucial to improving chemosensitivity and developing targeted therapy for inhibiting EMT.

Several studies have explored the potential of sphere formation, a well-established method for maintaining cells with stem cell-like properties [15–18]. In our previous study, we performed cDNA microarray analysis using adherent and sphere cells from the human BTC cell lines SNU1196 and SNU245 to evaluate the unique molecular patterns of BTC CSCs and identified 70 genes (1.2 > FC (fold change) in spheres and 2 > FC in BTC) [19]. Among the identified genes, *Transgelin-2 (TAGLN2)* was overexpressed in BTC and CSCs. TAGLN2, an actin-binding protein highly expressed by tumor cells, plays a crucial role in determining cell morphology and transformation [20] and has been implicated in various human malignancies [21–32]. The potential involvement of TAGLN2 in metastasis, either through direct interaction with cytoplasmic actin or induced expression of metastasis-related genes, has been reported in several studies [30, 33, 34]. A recent study exploring the role of *TAGLN2* in cancer demonstrated its association with multidrug resistance and metastasis in breast cancer, emphasizing the potential of *TAGLN2*-targeted therapies [35]. Furthermore, *TAGLN2* has been identified as an oncogene related to prognosis and immunity across various cancers [31], and its involvement in tumor proliferation and migration in colorectal cancer has also been documented [28].

In this study, we investigated the malignant behavior of TAGLN2 in BTC cells and analyzed its expression in patient tissue and serum. Suppressing *TAGLN2* expression by short hairpin RNAs (shRNA) decreased cell motility, tumorigenic ability, down-regulated CSC-related markers, and enhanced cell sensitivity to radiation or chemotherapeutics. In patient samples, TAGLN2 expression was enhanced in both cancer tissues and patient serums. Our data suggest that *TAGLN2* is a putative diagnostic marker and therapeutic target for BTC that targets EMT and CSCs.

## Materials and methods

### Cell culture

The human BTC cell lines SNU245, SNU308, SNU478, SNU869, SNU1079, and SNU1196 were purchased from the Korea Cell Line Bank (KCLB, Seoul, Korea). These cell lines were maintained in RPMI1640 (Invitrogen Gibco, Grand Island, NY, USA) supplemented with 10% fetal bovine serum (FBS; Hyclone, Logan, UT, US). NIH-3T3 mouse fibroblast cells were purchased from the American Type Culture Collection (ATCC Manassas, VA, USA) and maintained in DMEM (Invitrogen, Carlsbad, CA, USA) supplemented with 10% FBS. We previously established gemcitabine-resistant BTC cells by escalating doses of gemcitabine treatment in SNU-1196 cells, as described in a previous study [19]. Cells were maintained at 37 °C in a humidified incubator with 5% CO_2_.

### Reagents

Inhibitors and chemotherapy drugs including 5-FU, cisplatin, oxaliplatin, carboplatin, irinotecan, etoposide, erlotinib, crizotinib, and savolitinib were purchased from Selleck Chemicals (Houston, TX, USA); gemcitabine was supplied by Eli Lilly Korea (Seoul, Korea).

### Sphere-formation assay

Sphere formation assay was performed as described in a previous study [35]. Briefly, cells were trypsinized and resuspended at a density of 1 × 10^3^/well in D/F12 (Invitrogen Gibco) medium supplemented with 10 ng/mL epidermal growth factor (R&D Systems Inc., Minneapolis, MN, USA), 10 ng/mL basic fibroblast growth factor (R&D Systems Inc.), 1X insulin-transferring selenium (Invitrogen), 0.5% bovine serum albumin (Invitrogen), and 0.5% FBS in ultra-low attachment culture plates (Corning Inc., Corning, NY, USA). Adherent cultured cells, as a control, were seeded in culture dishes (Nalgene Nunc Intl, Rochester, NY, USA) with a sphere formation medium. After seven days, cells were collected and dissociated with Accutase (Sigma-Aldrich, St. Louis, MO, USA).

### Microarray analysis

Total RNA was extracted from adherent cells and cell spheres of SNU245 and SNU1196 cells using RNeasy Miniprep kits (Qiagen, Valencia, CA, USA). Microarray analysis was performed according to the kit’s protocol [19]. Briefly, double-stranded cDNA was prepared using 6 µg aliquots of total RNA, amplified using polymerase chain reaction (PCR), and labeled with biotin using an IVT labeling kit (Affymetrix, Santa Clara, CA, USA). The labeled cDNA was fragmented and hybridized to an Affymetrix GeneChip Human Genome U133 Plus 2.0 high-density oligonucleotide Array (Affymetrix). The microarrays were then washed using a GeneChip Fluidics Station 450 (Affymetrix) and scanned using a GeneChip Array Scanner 3000 7G (Affymetrix). Expression data were generated using Affymetrix Expression Console software version 1.1 using MAS5 algorithm normalization. The expression intensity data in the CEL file were normalized using the MAS5 algorithm to reduce noise.

### Silencing TAGLN2 expression using shRNA, small interfering RNA (siRNA)

To verify the effects of *TAGLN2* inhibition, a *TAGLN2* knockdown cell line was established by transfecting *TAGLN2* shRNA (targeting sequence: 5′-GTGCTATGTGAGCTCATTAAT-3′) or mock shRNA (targeting sequence: 5′-GGAATCTCATTCGATGCATAC-3′) plasmids (Sure Silencing shRNA plasmids; cat. 336,314 KH19252P) into SNU1196 cells using Lipofectamine 2000 (Invitrogen), followed by treatment with puromycin (2 µg/mL). Single colonies were picked, and TAGLN2 expression was assessed using western blotting. *TAGLN2* siRNA (Santa Cruz Biotechnology, Santa Cruz, CA; sc-106,633) comprising a pool of a target-specific siRNA (siTAGLN2) and control siRNA-A (Santa Cruz Biotechnology; sc-37,007) at 100 pmol/L in 150 nM medium were transfected into SNU1196/GR cells using Lipofectamine RNAiMAX (Invitrogen).

### Semiquantitative reverse-transcription PCR (RT-PCR)

Total RNA was extracted for RT-PCR using RNAeasy Mini kits (Qiagen), and single-stranded cDNA was synthesized using a Superscript II system (Invitrogen), according to the manufacturer’s instructions. The expression of *TAGLN2* was evaluated in gastric cancer spheres and control cells, and the *Actin beta* (*ACTB*) gene was used as the reference gene. The following primers were used for RT-PCR: *TAGLN2* forward primer: 5′-TAT GGC ATT AAC ACC ACT GA-3 ′; *TAGLN2* reverse primer: 5′-GGA TTC TCC TTG GAT TTC TT-3 ′; *ACTB* forward primer: 5′-GGC ATC CTC ACC CTG AAG TA-3′; *ACTB* reverse primer: 5′-GGG GTG TTG AAG GTC TCA AA-3′.

### Western blotting

Western blotting was performed as described in a previous study [19]. Briefly, cells were homogenized in lysis buffer (70 mM glycerophosphate, pH 7.2, 0.6 mM Na vanadate, 2 mM MgCl_2_, 1 mM EGTA, 1 mM DTT, 0.5% Triton X-100, 0.2 mM phenylmethylsulfonyl fluoride, and 1X complete protease inhibitor; Roche Applied Science, Nutley, NJ, USA), incubated on ice for 1 h, centrifuged at 12,000 rpm for 1 h at 4 °C, and the supernatant was collected. Recombinant TAGLN2 (rTAGLN2) was generated by AbFrontier (Seoul, Republic of Korea) by expressing the full-length *TAGLN2* sequence in the pET21a vector in BL21 cells for three days, and the cells were collected. Cells were washed with phosphate-buffered solution (PBS) and cultured with serum-free RPMI (Invitrogen Gibco) for two days for protein precipitation from culture media. The culture supernatant was collected, mixed with acetone, and stored at − 20℃ overnight. The mixture was centrifuged at 13,000 rpm for 30 min at 4 °C, and the pellet was collected and resuspended in lysis buffer. Patient blood samples were collected in 10 mL BD serum tubes, centrifuged at 4 °C for 20 min at 3,000 × g, and 3 µL of the supernatant serum samples were loaded. Primary antibodies against TAGLN2 (Sigma-Aldrich), N-cadherin (Abcam, Cambridge, UK), Snail, Nanog, JAG2, phospho-GSK3, phospho-AKT, phospho-MEK, MAPK, phospho-ERK (Cell Signaling Technology, Inc., Danvers, MA, USA), cMET, occludin, AKT, MEK, ERK, and GAPDH (Santa Cruz Biotechnology) were used. The secondary antibodies used were goat anti-mouse HRP and goat anti-rabbit immunoglobulin G (IgG)-HRP (Santa Cruz Biotechnology; 1:5000). Proteins were visualized using Super Signal® West Pico Chemiluminescent Substrate (Thermo Scientific, Rockford, IL, USA).

### Cell proliferation assay

The cell proliferation assay was performed using WST-1 reagent (Roche Applied Science) according to the manufacturer’s instructions. SNU-1196 cells transfected with TAGLN2 or control shRNA were seeded into 96-well plates at 1 × 10^3^/well in 100 µL culture medium. After 48 h, WST-1 (10 µL) was added to each well and incubated at 37 °C for 1 h. Absorbance was measured at 450 nm using a VersaMax ELISA microplate reader (Molecular Devices, USA).

### Migration and invasion assays

Migration and invasion assays were performed as described in a previous study [35]. For the migration assay, cells were detached and suspended at 1 × 10^5^ cells/mL in serum-free media and plated at a density of 1 × 10^4^ cells/well in 24-well Transwell plates (Costar, Bethesda, MD). For the invasion assay, the upper chamber was pre-coated with Matrigel (1:4 diluted with serum-free medium; BD Biosciences), and cells were seeded at a density of 1 × 10^4^ cells/well. The bottom chamber was filled with the culture medium containing NIH-3T3 fibroblasts. The cells were incubated for 24 and 72 h for migration and invasion assays, respectively. After incubation, cells were fixed with 5% glutaraldehyde for 30 min and stained with 0.1% crystal violet. The cells were completely removed from the upper surface of the membrane using a moist cotton swab. The migrated and invaded cells were counted and photographed under a microscope at 100× magnification. All assays were performed in triplicates.

### Colony formation assay and cell irradiation

Colony formation assay was performed as described in a previous study [35]. Briefly, 3 × 10^2^ cells were suspended in 0.5 mL Difco Noble Agar (0.3%; Becton Dickinson Company, Sparks, MD) supplemented with sphere formation medium and plated in 24-well plates containing 0.6% agar. All samples were plated in triplicates. Cells were incubated for 2–3 weeks in a humidified incubator with 5% CO_2_ at 37 °C, and the sphere formation medium was changed every alternative day. Radioresistance was analyzed by subjecting cells to 0–8 Gy of ionizing radiation (Gammacell 3000 Elan, MDS Nordion, Ottawa, ON, Canada), followed by a colony formation assay.

### Cell viability assay

Cells were seeded at in 96-well plates and treated with inhibitors and chemotherapy drugs at various concentrations for 72 h. Cell viability was evaluated using the 3-(4,5-dimethylthiazol-2yl)-2,5-diphenyltetrazolium bromide (MTT) assay (AMRESCO, Solon, OH, USA) [19]. The half-maximal inhibitory concentration (IC_50_) was analyzed relative to that of the DMSO control. Values are shown as the mean of triplicate wells from three independent experiments for each drug concentration. Absorbance was measured at 570 nm using a VersaMax ELISA microplate reader (Molecular Devices). Dose-effect data for individual drugs and their combinations were analysed for synergism using CompuSyn software (http://www.combosyn.com/).

### Tumorigenicity assays

Tumorigenicity assay was performed as described in a previous study [19]. Briefly, cells were washed with PBS, suspended in serum-free RPMI (Invitrogen Gibco) and Matrigel (BD Biosciences PharMingen; 1:1 volume), and subcutaneously injected into the right flank of six-week-old female BABL/c nude mice (Orient Bio, Seongnam, Korea) [19]. The tumor volume was calculated as V (mm^3^) = (A^2^×B)/2, where A is the diameter perpendicular to the largest dimension, B. After 14–16 weeks, the mice were sacrificed in a CO_2_ chamber, and tumor tissues were fixed in 4% paraformaldehyde. For histological evaluation, tissue samples were embedded in paraffin and stained with hematoxylin and eosin (H&E). All animal experiments were approved by the Committee for the Care and Use of Laboratory Animals of Yonsei University College of Medicine, and the study is reported in accordance with ARRIVE guidelines(https://arriveguidelines.org).

### Patients

We analyzed 41 human BTC tissue samples obtained from surgical resections and 139 human blood samples from 89 patients with BTC, 10 patients with biliary stones, and 40 normal controls at Severance Hospital, Yonsei University College of Medicine. The Ethical Committee for Clinical Research of the Institutional Review Board of Severance Hospital, Yonsei University College of Medicine, Seoul, Korea, approved the study protocol (IRB approval code:4-2011-0625; November 24, 2011). All procedures involving human participants were performed in accordance with the ethical standards of the Institutional Research Committee and the 1964 Declaration of Helsinki and its later amendments or comparable ethical standards. Written informed consent was obtained from all subjects. Information regarding patient demographics and clinical data were obtained from electronic medical records, including age at diagnosis, sex, tumor stage at diagnosis, and serum carcinoembryonic antigen (CEA) levels. Tumors were staged according to the 7th edition of the American Joint Committee on Cancer (AJCC) staging classification.

### Immunohistochemical and immunofluorescence staining

Immunohistochemical and immunofluorescence staining were performed as described in a previous study [35]. For immunohistochemical staining, tissue slides were deparaffinized in xylene and rehydrated in graded alcohol. Endogenous peroxidase activity was blocked with 0.3% (v/v) hydrogen peroxide in methanol. Antigen retrieval was performed by microwaving the slides in a sodium citrate buffer (0.01 M, pH 6.0) for 5 min. To block nonspecific staining, sections were incubated with 10% (v/v) normal donkey serum for 1 h; then, the sections were incubated with appropriate antibodies overnight at 4 °C. Subsequent reactions were performed using Envision kits (Dako Cytomation California, Inc., Carpinteria, CA, USA) following the manufacturer’s instructions. Immunoreactions were developed with the DAKO Liquid diaminobenzidine substrate-chromogen system (DAB+) and counterstained with Harris hematoxylin (Sigma-Aldrich). The reaction was subsequently carried out with an LSAB + Kit (Dako), and sections were counterstained with Mayer’s hematoxylin, dehydrated, and observed under a BX51 microscope (Olympus, Tokyo, Japan). For immunofluorescence staining, tissue slides were visualized using Cy5-goat anti-rabbit IgG dissolved in an antibody diluent and incubated for 30 min at room temperature [35]. Between each step, three washing steps of 5 min each were performed on a rocking platform using PBS. The slides were cover-slipped using a mounting medium for observing fluorescence with DAPI (Vecta shield H-1200; Vector Laboratories, Inc. Burlingame, CA, USA). Primary antibodies against TAGLN2 (Sigma-Aldrich), alpha-smooth muscle actin (α-SMA), fibroblast-associated protein (FAP), and cytokeratin-7 (CK-7) (Santa Cruz Biotechnology) were used.

### Statistical analysis

Categorical data were analyzed using χ^2^ and Fisher’s exact tests. Student’s t-test and Mann–Whitney U test were used for continuous variables. Survival was estimated and compared using Kaplan–Meier analysis with a log-rank test. Serum TAGLN2 and CA19–9 levels were compared between patients with benign and biliary cancer using the non-parametric Kruskal–Wallis test. The cut-off value, receiver operating characteristic (ROC) curve, area under the ROC curve (AUC), and 95% confidence intervals (CI) were determined. All statistical analyses were performed using IBM SPSS Statistics for Windows (version 25.0; IBM Corp., Armonk, NY). A *P*-value of < 0.05 was considered statistically significant.

## Results

### TAGLN2 is differentially expressed in BTC sphere cells

**Fig. 1:**
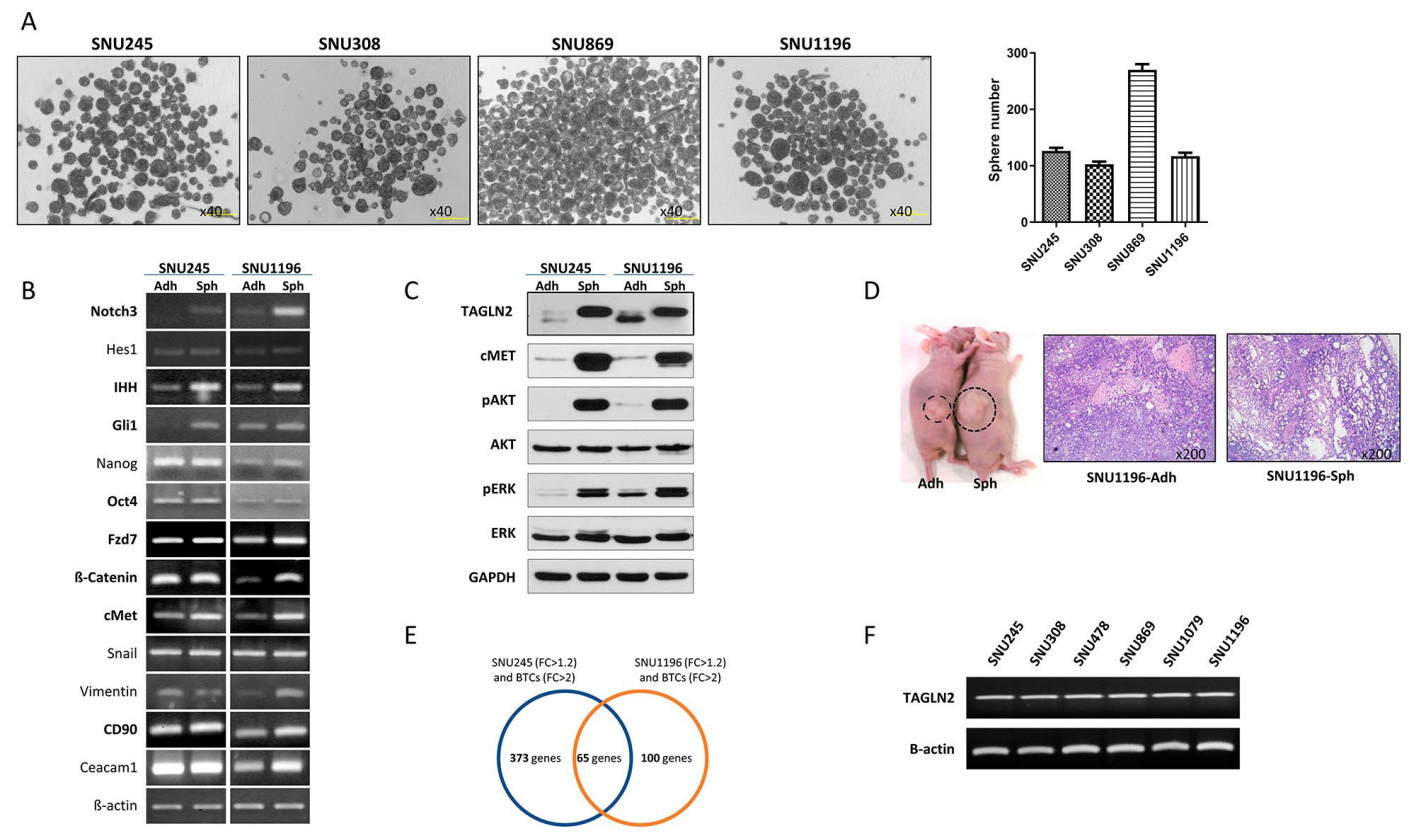
Expression of *Transgelin-2* (TAGLN2) in the biliary tract cancer cell (BTC)-spheres. (**A**) Sphere-formation assays of BTC cell lines, including SNU245, SNU308, SNU869, and SNU1196. The cells were maintained in growth media and plated onto culture dishes or ultra-low attached plates in sphere media. (**B**) Differentially expressed genes and (**C**) proteins analyzed in sphere-cultured cells in SNU245 and SNU1196. (**D**) Tumorigenesis was performed using adherent and sphere-cultured SNU1196 in vivo. (**E**) Differentially expressed genes were analyzed using microarray analysis; 65 genes were overexpressed in both sphere cultured SNU245 and SNU1196 cells (Fold change, FC > 1.2) as well as in biliary tract cancer patient tissues (FC > 2); *TAGLN2* was selected from the microarray analysis. (**F**)*TAGLN2* expression in all BTC cell lines analyzed using PCR. In B, C, D, and F: Adh, adherent cells; Sph, sphere cells

Estimation of the sphere-forming capacities of different human BTC cell lines (3 × 10^3^/well) revealed that SNU245, SNU308, SNU869, and SNU1196 reproducibly formed spheres (126.33 ± 5.51, 102.67 ± 4.62, 270.0 ± 10.1, and 117.0 ± 6.08 per well, respectively) in vitro, whereas SNU478 partially formed and SNU1079 did not form spheres and remained as aggregated cell clusters (Fig. 1A, Supplementary Fig. 1A). Analysis of differentially expressed genes (DEGs) revealed the upregulation of Hedgehog signaling pathway-associated genes (*IHH* and *Gli1)*, Wnt signaling pathway-associated genes (*FZD7*, *beta catenin*), other stemness-associated genes (*Notch3*), epithelial-to-mesenchymal transition-associated genes (*cMET* and *vimentin*), and liver cancer stem cell surface marker (*CD24* and *CD90*) in sphere-cultured SNU245, SU308, SNU869, and SNU1196 cells (Fig. 1B, Supplementary Fig. 1B). Concordantly, western blot analysis revealed elevated expression of cMET, phospho-AKT, and phospho-ERK in the spheres (Fig. 1C, Supplementary Fig. 1C). The injection of adherent and sphere-cultured SNU1196 cells (1 × 10^3^) into BABL/c nude mice showed larger tumors with poorer differentiation in the sphere-cultured cells than those in the adherent cells at 12 weeks which indicated the enhanced tumorigenic ability of sphere-cultured cells in vivo (Fig. 1D, Supplementary Fig. 1D). In addition, the DEGs were further confirmed by microarray analysis. Subsequently, we focused on the DEGs upregulated in spheres in both cell lines as well as in tissues obtained from patients with BTC (*n* = 6). Among these genes, *TAGLN2* was upregulated 1.7-, 1.5-, and 7.7-folds in SNU245, SNU1196, and BTC tissues, respectively (Fig. 1E). These results were further confirmed by the elevated expression of TAGLN2 in the spheres determined using western blot analysis (Fig. 1C, Supplementary Fig. 1C). Moreover, *TAGLN2* was constitutively expressed in all BTC cell lines, with differences in the basal expression levels (Fig. 1F).

The IC_50_ for crizotinib ranged from 0.31 nM to 3.72 µM, while that of savolitinib was 112 µM and higher in BTC cells. Treatment of the cells with cMET inhibitors, crizotinib and savolitinib, altered the cell viability, particularly at concentrations higher than those of respective IC_50_ values (Supplementary Fig. 2A). Furthermore, exposure of the spheres to both inhibitors (at 50 nM) reduced the number and size of the spheres (Supplementary Fig. 2B). These findings provide evidence that the targeted inhibition of cMET specifically impedes cell viability in sphere-cultured cells.

### Suppression of TAGLN2 expression reduces the proliferation and motility of BTC cells in vitro and in vivo

**Fig. 2:**
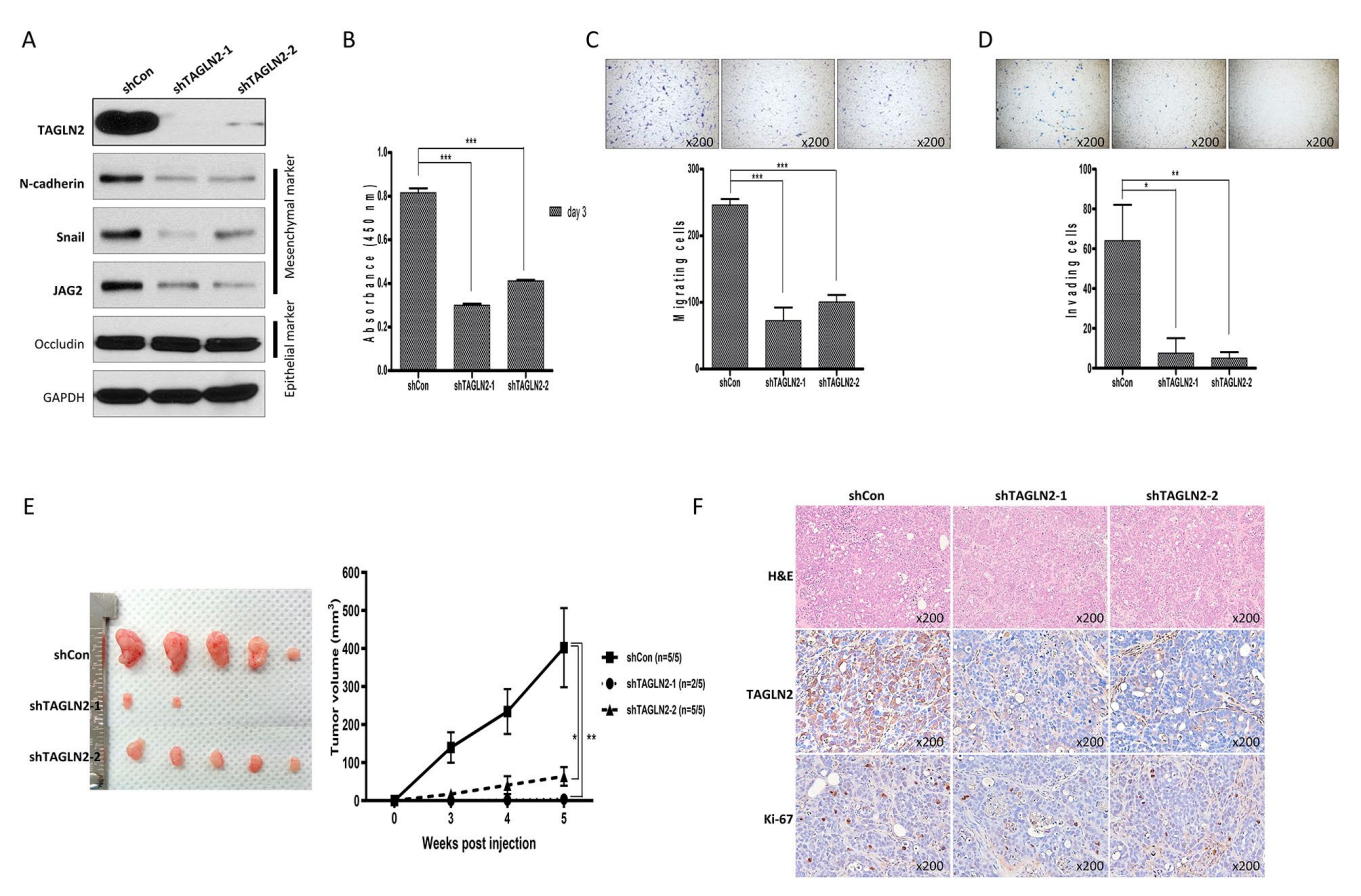
Suppressing *TAGLN2* expression reduces cell proliferation, migration, and tumorigenic ability. (**A**) The effect of *TAGLN2* suppression on the expression of EMT-associated proteins, including mesenchymal markers N-cadherin, Snail, and JAG2 and epithelial marker occludin. (**B–D**) *TAGLN2* suppression by shRNA reduces (**B**) cell proliferation, (**C**) migration, and (**D**) invasion; ***, *P* < 0.001 shTAGLN2-1- and shTAGLN2-2-transfected vs. control vector-transfected (shCon) SNU1196 cells. (**E**) Injection of shTAGLN2-1, shTAGLN2-2 cells suppressing *TAGLN2* expression into mice reduces the tumor mass in treated groups compared to the shCon group; *n* = 5 in each group, Data represents mean ± SEM. (**F**) The expression of Ki67, a cell proliferation marker in *TAGLN2*-suppressed mice tumor tissues

To evaluate the functional role of *TAGLN2*, we suppressed *TAGLN2* expression in SNU1196 using shRNA. The evaluation of the effect of *TAGLN2* suppression on the expression of EMT-associated proteins demonstrated their considerable downregulation, including those of the mesenchymal markers N-cadherin, Snail, and JAG2; however, the expression of occludin did not change (Fig. 2A). After shTAGLN2-1 and shTAGLN2-2 treatment, SNU-1196 cell proliferation was significantly suppressed by an average of approximately 63% (36.84 ± 0.31%) and 50% (50.48 ± 0.19%), respectively. Concurrently, treatment with shTAGLN2-1 and shTAGLN2-2 also significantly reduced the migration (by 29.51 ± 1.94% and 40.88 ± 1.03%, respectively) and invasion (by 11.71 ± 0.42% and 7.81 ± 0.17%, respectively) of SNU1196 cells compared to control vector-transfected (shCon) SNU1196 cells (Fig. 2B–D). TAGLN2 expression also altered tumorigenicity in vivo. The injection of shTAGLN2-1 and shTAGLN2-2 to mice resulted in significantly undersized tumor mass in the shTAGLN2-1 (*n* = 5, 3.75 ± 5.13 mm^3^, mean ± SEM) and shTAGLN2-2 (*n* = 5, 63.81 ± 24.5 mm^3^) groups, compared to that in the shCon group (*n* = 5, 402 ± 233.13 mm^3^) (Fig. 2E). Immunohistochemical analysis using antibodies against TAGLN2 and Ki-67, a proliferation marker, on xenograft tumors demonstrated decreased Ki-67 expression in shTAGLN2-1- and shTAGLN2-2-treated groups compared to that in the shCon-treated group (Fig. 2F, Supplementary Fig. 3).

### Suppressing *TAGLN2* expression reduces cancer stem cell characteristics

**Fig. 3:**
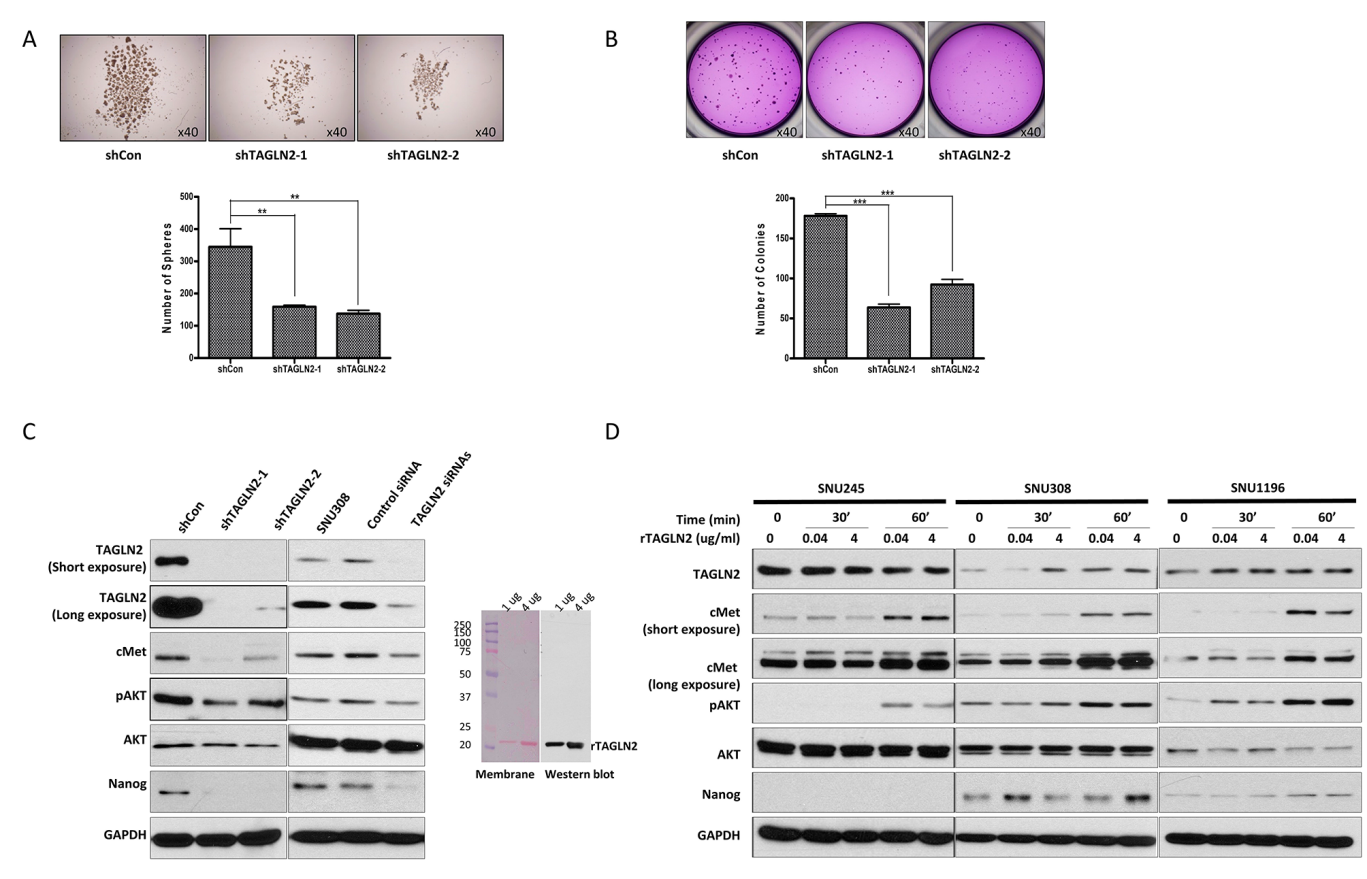
Suppressing *TAGLN2* expression reduces cancer stem cell features. (**A**) Sphere formation assay. Cells with suppressed *TAGLN2* (shTAGLN2-1, -2) show a reduced number and size of spheres compared to the control vector-transfected cells. (**B**) Colony forming assay to analyze the clonogenic capacity. The colonies in shTAGLN2-1- and shTAGLN2-2-transfected SNU1196 cells were increased compared to those in control vector-transfected cells (*P* < 0.001). (**C**) The expression of stemness-associated markers, including cMET, AKT, and Nanog, assessed using western blot analysis in SNU1196 and SNU308 cells with suppressed *TAGLN2* expression achieved by either shRNA or siRNA. (**D)** SNU245, SNU308, and SNU1196 cells treated with recombinant TAGLN2 (rTAGLN2) protein at 0, 0.04, and 4 µg/mL for 30 or 60 min. An increase in cMET, AKT, and Nanog is observed in a dose- and time-dependent manner by rTAGLN2 treatment in both cell lines

Sphere formation assay demonstrated that the number and size of spheres were decreased two-fold in cells with suppressed *TAGLN2* (shTAGLN2-1, -2) compared to those in control vector-transfected cells (Fig. 3A). The colony-forming assay revealed a significant reduction in clonogenic capacity in shTAGLN2-1 and − 2 transfected cells by 35.73 ± 1.54% and 51.82 ± 2.39%, respectively, compared to the shCon-transfected cells (*P* < 0.001; Fig. 3B). Furthermore, the expression of stemness-associated markers, including c-MET, phospho-AKT, and Nanog, decreased in SNU1196 and SNU308 cells, in which *TAGLN2* was suppressed by either shRNA or siRNA (Fig. 3C). To validate whether *TAGLN2* expression regulates cMET, phospho-AKT, and Nanog, SNU245, SNU308, and SNU1196 cells were treated with rTAGLN2 protein at 0, 0.04, and 4 µg/mL for 30 or 60 min. An increase in c-MET, AKT, and Nanog levels was observed in a dose- and time-dependent manner following rTAGLN2 treatment in both cell lines, in contrast to the suppression of *TAGLN2* (Fig. 3D).

### Suppressing *TAGLN2* enhances sensitivity to radiation and chemotherapeutics

**Fig. 4:**
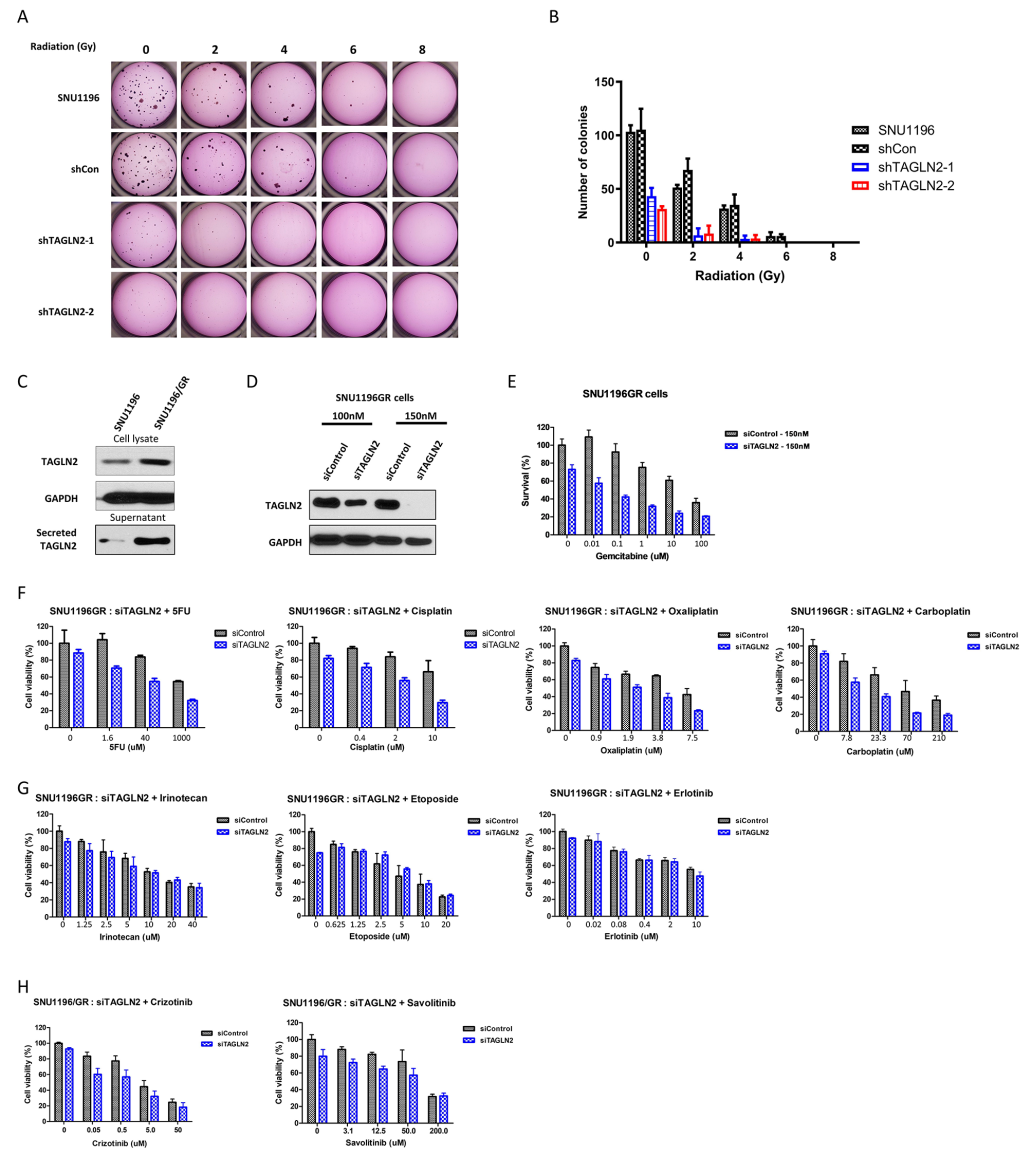
Suppressing *TAGLN2* elevates sensitivity to chemodrugs. (**A, B**) Irradiation (0, 2, 4, 6, and 8 Gy) reduces the number of colonies and the surviving fractions at different doses in cells in which *TAGLN2* is suppressed using shRNA (shTAGLN2-1, -2) compared to control vector-transfected cells. (**C**) TAGLN2 expression in gemcitabine-resistant SNU1196 (SNU1196/GR). (**D**) TAGLN2 expression silenced by 150 nM siRNA in 1196/GR cells for chemosensitivity analysis. (**E)** Silencing *TAGLN2* expression enhances sensitivity to gemcitabine. (**F**) Combined use of *TAGLN2* siRNA with chemodrugs, including 5-FU, cisplatin, oxaliplatin, and carboplatin, enhances chemosensitivity. (**G**) Combined use of *TAGLN2* siRNA with chemodrugs, including irinotecan, etoposide, and erlotinib, has no effect on chemosensitivity. (**H**) Combined use of *TAGLN2* siRNA with cMET inhibitor crizotinib and savolitinib enhances chemosensitivity

To analyze radioresistance, the cells were irradiated (0, 2, 4, 6, and 8 Gy), and the number of colonies was compared using a colony formation assay. Irradiation drastically reduced the number of colonies in *TAGLN2*-suppressed cells. The numbers of colonies were 43.33 ± 7.64, 6.67 ± 6.51, and 3.33 ± 3.06; 31.33 ± 2.52, 8.33 ± 7.37, and 3.67 ± 3.22; 105.33 ± 19.50, 67.67 ± 10.69, and 35.00 ± 9.50 in shTAGLN2-1, shTAGLN2-2, and shCon-transfected cells after 0, 2, and 4 Gy irradiation, respectively (Fig. 4A–B). At 6 Gy irradiation, no colonies were formed in cells with suppressed *TAGLN2* expression, whereas colonies were observed in control vector-transfected cells. The numbers of colonies and surviving fractions are shown in Supplementary Fig. 4.

We then used previously established gemcitabine-resistant BTC cells (SNU1196/GR) to mimic cases in which first-line chemotherapy failed owing to the development of drug tolerance. SNU1196/GR cells have a gemcitabine IC_50_ of 2.291 µM, approximately 60-fold higher than that of parental cells (SNU1196 cells, IC_50_ = 0.038 µM) [19]. These cells showed elevated expression of the ATP-binding cassette (ABC) transporter ABCG2, proto-oncogenes c-Met and AKT, EMT marker N-cadherin, and tumor-initiating cell surface marker aldehyde dehydrogenase (ALDH) both in vitro and in vivo. TAGLN2 was significantly overexpressed in cell lysates and supernatants of SNU1196/GR cells (Fig. 4C). We analyzed cell viability after serial doses of gemcitabine treatment in SNU1196/GR and SNU1196/GR cells in which *TAGLN2* expression was silenced by siRNAs (Fig. 4D). Cell viability was decreased by approximately 30% in siTAGLN2-transfected cells, which was further decreased to ∼ 50% in gemcitabine-treated (0.01–10 µM) cells compared to control siRNA-transfected SNU1196/GR cells (Fig. 4E). We evaluated whether the expression of TAGLN2 affects the chemosensitivity of other chemodrugs, including anti-metabolites (5-FU), platinum-based anti-cancer drugs (cisplatin, oxaliplatin, and carboplatin), topoisomerase inhibitors (irinotecan and etoposide), an EGFR inhibitor (erlotinib), and cMET inhibitors (crizotinib and savolitinib) in SNU1196/GR cells. Each of the drugs tested, including gemcitabine, 5-FU, cisplatin, oxaliplatin, carboplatin, irinotecan, etoposide, crizotinib, and savolitinib was compared with the control vector and siTAGLN2 transfection (150 nM each), and cell viability was analyzed using CompuSyn software to calculate the combination index (CI) to analyze the additive and synergistic effects of standard chemotherapy drugs in combination with siTAGLN2 transfection at different concentrations (Fig. 4F–H). CI < 1, CI = 1, and CI > 1 indicated synergistic, additive, and antagonistic effects, respectively. In SNU1196/GR cells, siTAGLN2 transfection had both additive and synergistic effects with gemcitabine, 5-FU, cisplatin, oxaliplatin, and carboplatin treatment, as evidenced by the CI values. siTAGLN2 exhibited synergistic cytotoxicity at different 5-FU concentrations (1.6–10,000 µM), with CI values of 0.012 to 0.030. Moreover, 5-FU concentration greater than 1000 µM was required to achieve 50% inhibition, while approximately 40 µM 5-FU was required to achieve the same IC_50_ with siTAGLN2 transfection (Supplementary Table 1).

### TAGLN2 is overexpressed in the blood samples from patients with BTC

Western blot analysis of the supernatant of BTC cell lines SNU1196 and SNU1196/GR, which are resistant to gemcitabine, revealed higher expression of TAGLN2 in SNU1196/GR cells than that in SNU1196 cells (Fig. 4C). To confirm the potential of TAGLN2 as a secretory biomarker for BTC, we examined TAGLN2 in the plasma samples of patients using western blot analysis (Supplementary Fig. 5). The baseline characteristics of the patients are shown in Supplementary Table 2. A total of 139 participants [89 patients with BTC, 10 patients with biliary stones, and 40 normal controls] were included in the western blot analysis. Among the patients with BTC, 29 (32.6%) had intrahepatic cholangiocarcinoma (IHCC), 28 (31.5%) had perihilar cholangiocarcinoma (PHCC), and 32 (36.0%) had distal common bile duct (CBD) cancer. The clinical stages at the time of blood sampling were stage I in 6 (6.7%), stage II in 35 (39.3%), stage III in 29 (32.6%) of stage III), and stage IV in 19 (21.3%) patients.

**Fig. 5:**
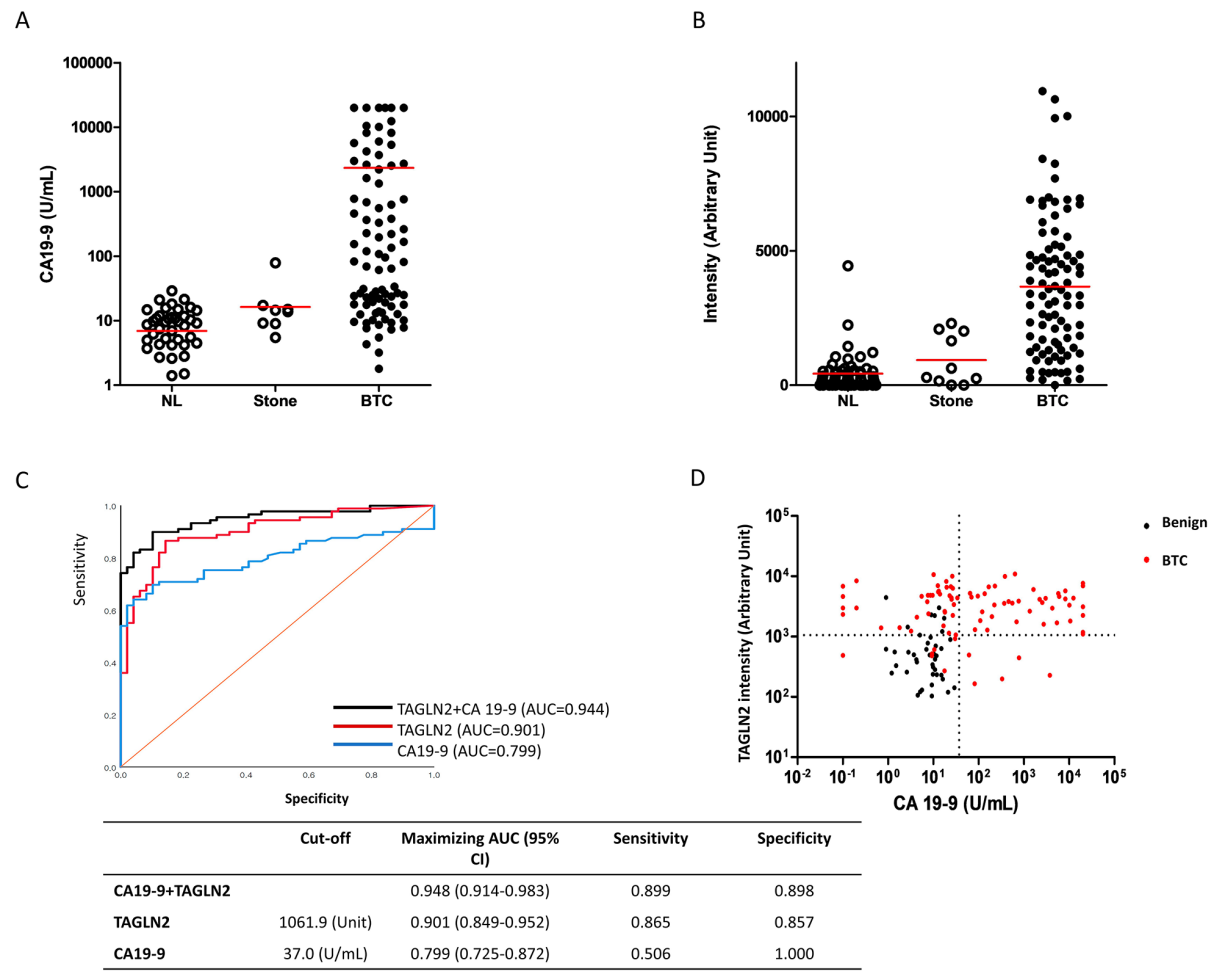
TAGLN2 is overexpressed in the blood from patients with biliary tract cancer. (**A**) Dot plot for the serum level of CA19–9 in normal control and patients with biliary stone and BTC. (**B**) Dot plot for the serum TAGLN2 expression in western blot converted into an arbitrary unit by densitography using ImageJ software in normal control and patients with biliary stone and BTC. (**C**) ROC curves for TAGLN2, CA19–9, and their mathematical combination of patients with BTC versus those with benign diseases. (**D**) Distribution of TAGLN2 and CA 19 − 9 levels in patients with benign diseases (black dot) and BTC (red dot). The optimal cut-off levels of CA 19 − 9 and TAGLN2 were 37 U/mL and 1061.9 arbitrary unit, respectively. Thirty-seven CA 19 − 9 negative patients with BTC (red dots in Left upper quadrant) were diagnosed using TAGLN2 (diagnostic yield, 84.1% (37/44 cases)

To evaluate the diagnostic significance of TAGLN2 compared to that of CA19–9, serum CA19-9 levels were compared between normal controls, patients with biliary stones, and BTC, which showed no significant difference in the mean levels of serum CA19-9 between normal control (9.0 ± 6.3 U/mL) and patients with biliary stone (12.1 ± 6.7 U/mL) (*P* = 0.194, Fig. 5A). Patients with BTC also showed significantly higher serum CA19-9 levels than those with benign cancers (9.6 ± 6.4 vs. 2417 ± 5377 U/mL; *P* = 0.0021, Fig. 5A). The band intensity of TAGLN2 obtained by western blotting was converted to arbitrary units by densitometry using ImageJ software. As shown in Fig. 5B, mean levels of serum TAGLN2 expression did not differ between normal control (538 ± 776 arbitrary unit) and patients with biliary stone (953 ± 1087 arbitrary unit, *P* = 0.171). Patients with BTC showed significantly higher serum TAGLN2 expression than those with benign diseases (622 ± 851 vs. 3710 ± 2568 arbitrary units, *P* < 0.0001). These data confirm that TAGLN2 is a secretory biomarker protein detectable in human blood that is upregulated in patients with BTC compared to that in healthy individuals or those with benign diseases.

To evaluate the diagnostic performance of serum TAGLN2 and CA19–9 levels in differentiating benign and BTC samples, the AUC was calculated using ROC curves. For TAGLN2, the AUC was 0.901 (95% CI: 0.849–0.952), and that of CA19–9 was 0.799 (95% CI: 0.725–0.872), with a significant difference (*P* = 0.026, Fig. 5C). With the best cutoff value of 1061.9 arbitrary unit, the sensitivity and specificity of TAGLN2 for differentiating BTC from benign conditions were 0.865% and 0.857%, respectively. For CA19–9, the sensitivity and specificity were 0.506 and 1.000, respectively, at a cut-off value of 37 U/mL. The ROC AUC of the combination of TAGLN2 and CA19–9 (0.948; 95% CI: 0.914–0.983)was significantly higher than that of CA19-9 alone (*P* < 0.0001, Fig. 5C). Figure 5D shows the distribution of TAGLN2 and CA 19 − 9 levels in patients with benign diseases and BTC. The number of patients with BTC who showed high TAGLN2 and normal CA 19 − 9 levels was 37 out of 44, with a diagnostic yield of 84.1%. These results indicate that TAGLN2 is a potential diagnostic biomarker of BTC.

### TAGLN2 is highly expressed in biliary cancer tissues and its expression in stroma is associated with patients’ survival

**Fig. 6:**
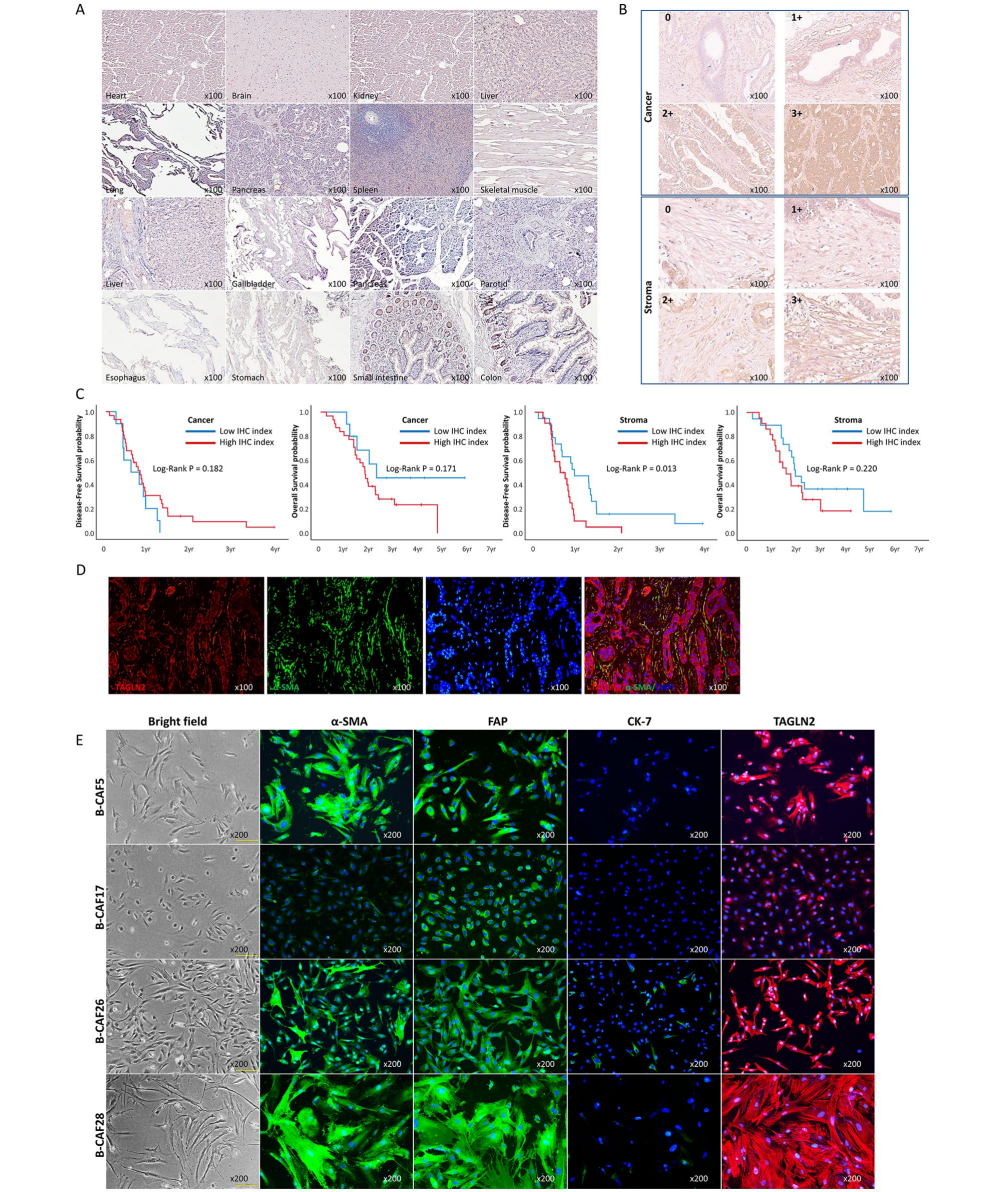
TAGLN2 expression in patient tissues and cancer-associated fibroblasts. (**A**) TAGLN2 expression in normal human tissues assessed using IHC. (**B**) Representative images show the expression of TAGLN2 in cancer cells and the stroma portion of BTC surgical tissues. (**C**) The DFS and OS by Kaplan–Meier analysis comparing low- and high-TAGLN2 IHC index in cancer and stroma, respectively. (**D**) IF findings in BTC tissue show overexpression of TAGLN2 in the stroma of BTC tissue, and its location coincided with α-SMA expression. (**E**) TAGLN2 expressed in the CAFs from BTC assessed using IF. Typical morphology of fibroblast in bright fields, expression of α-SMA and FAP, and non-expression of the epithelial marker as CK-7 in the IF study are shown

hTAGLN2 is weakly expressed in the small intestine and colon; however, its expression is rarely observed in other major organs. Therefore, prior to TAGLN2 expression analysis in BTC tissues, the expression of TAGLN2 in normal organ tissues was analyzed using immunohistochemistry (IHC; Fig. 6A, Supplementary Fig. 6A). Patient cancer tissues, along with their adjacent normal tissues, were also analyzed, and a comparably high expression was observed in the cancer tissues (Supplementary Fig. 6B). Subsequently, TAGLN2 expression in 41 surgical samples of human BTC using IHC in cancer cells and stroma around cancer was evaluated. The intensity score was defined as no staining (0), light brown (1), brown (2), or dark brown (3); the percentage score was defined as < 10% (0), 10–25% (1), 25–50% (2), 50–75% (3), or > 75% (4) (Fig. 6B, Supplementary Fig. 7). The IHC index was calculated by multiplying intensity and percentage scores. Patients were divided into low and high IHC index groups based on TAGLN2 expression in the cancerous and stromal portions. Patients with an IHC index > 7 were classified into the high-IHC group, and those with scores < 7 were classified into the low-IHC group.

Baseline characteristics of cancer and stroma IHC indices are described in Supplementary Tables 3 and 4. Among the 41 surgical samples, most presented a high cancer IHC index (*n* = 31, 75.6%), and no significant differences were observed in baseline characteristics between the low- and high-cancer IHC groups. In survival analysis using the Kaplan–Meier plot, no significant differences were observed between low- and high-cancer IHC groups in disease-free survival (DFS) (7.3 vs. 9.9 months, *P* = 0.182) and overall survival (OS) (26.9 vs. 21.4 months, *P* = 0.171). Evaluation of TAGLN2 expression in stromal tissues identified 22 patients (53.7%) in the high stroma IHC group and 19 (46.3%) in the low stroma IHC group. No significant differences between the low- and high-stroma IHC groups were observed in the baseline characteristics. Survival analysis showed significant prolonged DFS in the low stroma IHC group compared to that in the high stroma IHC group (11.5 vs. 7.4 months, *P* = 0.013). The OS was not significantly different between the low- and high-stroma IHC index groups (23.4 vs. 19.0 months, *P* = 0.220) (Fig. 6C).

To evaluate TAGLN2 expression in the stroma of surgical tissue, we performed an immunofluorescence (IF) study of surgical tissues with α-SMA, a representative marker of cancer-associated fibroblasts (CAFs), and TAGLN2. In the stroma of cancer tissues, TAGLN2 was overexpressed, and its localization coincided with α-SMA expression (Fig. 6D, Supplementary Fig. 8A). IF analysis confirmed that TAGLN2 was expressed in CAFs from patients with BTC (Fig. 6E, Supplementary Fig. 8B). CAF was characterized by typical morphology in bright fields, expression of representative CAF markers such as α-SMA and FAP, and non-expression of the epithelial marker, CK-7. These findings suggest that TAGLN2 is expressed in CAFs of BTC, and its overexpression is related to patient survival.

## Discussion

CSCs are a subset of cells within a tumor that possess the unique ability to self-renew, resist chemotherapy, and initiate tumor growth. The discovery of CSCs has led to a new understanding of tumorigenesis and the development of new treatments. Targeting CSCs, in addition to conventional chemotherapy targeting cancer cells, is crucial for better treatment outcomes because a small subpopulation of CSCs remains resistant to chemotherapy and gives rise to recurrent cancer.

Recently, *TAGLN2* has emerged as a biomarker that plays an essential role in developing various types of cancer [36]. Alteration of *TAGLN2* expression has been noted in different types of cancer at both the transcriptional and translational levels, and cancer cell proliferation, invasion, and metastasis might be inhibited by suppressing *TAGLN2*. Tumorigenesis and tumor development may be correlated with the deregulation of *TAGLN2*. Tumor size, clinical stage, histological neural invasion, and lymph node metastasis are closely associated with TAGLN2 in bladder cancer [37], colorectal cancer [38, 39], esophageal cancer [24], and gastric cancer [40]. Moreover, cancer cell proliferation and EMT, which are responsible for cancer development, dissemination, and resistance to chemotherapy, are decreased by the downregulation of TALGN2 in breast cancer [41], renal cell carcinoma [42], cervical cancer [43], and head and neck squamous cell carcinoma [22].

Previous studies have suggested that overexpression of TAGLN2 is a potential cause of chemoresistance by increasing EMT properties; however, the mechanism of cancer development and chemoresistance by TAGLN2 has not yet been identified. Recent studies focus on the correlation of TAGLN2 with signaling, including transforming growth factor-beta (TGF-β)/FoxM1 [25], TGF-β/SMAD4 [44], IGF1R/phosphoinositide 3-kinase (PI3K)/Akt [45], and PI3K/phosphatase and tensin homolog (PTEN)/Akt pathways [41, 46–48]. In this study, we demonstrated that the suppression of *TAGLN2* altered not only the expression of EMT-associated proteins, including N-cadherin, Snail, and JAG2, but also those of cMET, AKT, and Nanog in a dose- and time-dependent manner following rTAGLN2 treatment.

Several studies have attempted to overcome chemoresistance by targeting TAGLN2-related signal pathways. SB-T-121,205, a next-generation taxoid, shows antitumor activity by inhibiting the TAGLN2 and PI3K/Akt pathways in human breast cancer cells [49]. In paclitaxel-resistant breast cancer cells, salvianolic acid A downregulates the expression of TAGLN2 by activating the PI3K/Akt pathway, restoring chemoresistance to paclitaxel [41]. Here, we inhibited TAGLN2 expression in gemcitabine-resistant BTC cells and restored their chemosensitivity to gemcitabine and other chemotherapeutic drugs, including 5-FU, cisplatin, oxaliplatin, and carboplatin (Fig. 4). Moreover, silencing TAGLN2 in gemcitabine-resistant BTC cells showed both additive and synergistic effects on the therapeutic efficacy of gemcitabine, 5-FU, cisplatin, oxaliplatin, and carboplatin, suggesting the combination therapy of anti-TAGLN2 and a cytotoxic drug as a potential therapeutic strategy to overcome chemoresistance in BTC cells.

In the patient tissues, TAGLN2 is expressed in cancer cells with an overall high expression intensity, making it challenging to analyze its correlation with prognosis. However, it is rarely expressed in normal tissues. TAGLN2 suppression inhibited cancer cell proliferation and reduced resistance to conventional anti-cancer drugs; therefore, TAGLN2 can be applied to most patients with cholangiocarcinoma, suggesting that it is a novel therapeutic target with fewer off-target effects.

Previously, several markers expressed in CAF were reported as prognostic factors associated with the survival of BTC. A previous study has shown that the high expression of IL-33 in both cancer cells and stromal CAFs is associated with better two-year survival of patients with BTC [50]. The matricellular protein periostin expressed in α-SMA + CAFs was reported as a poor prognostic factor in post-resected BTC [51]. Another marker, stromal cell-derived factor-1, associated with tumor fibrogenesis and EMT, has also been shown to be correlated with reduced median survival in patients with BTC [52]. The findings of the present study demonstrated that TAGLN2 expression in patient tissues was increased in stromal tissues around cancer cells and that stromal expression of TAGLN2 was related to patient prognosis. IF analysis of the patient tissues confirmed that the expression of TAGLN2 coincided with that of α-SMA, suggesting that TAGLN2 is expressed in CAF. Furthermore, the IF of CAF derived from patients with cholangiocarcinoma confirmed the expression of TAGLN2 in CAFs. TAGLN2 has been reported as a myCAF marker [53]; in this study, we report TAGLN2 expression in CAF and its association with CAF from BTC. Even though our findings are based on a few patient samples, increased TAGLN2 expression in CAFs was associated with poor prognosis in patients with BTC. Nevertheless, further studies are required to understand the effect of TAGLN2 on the crosstalk between CAF and cancer cells.

The present study demonstrated that serum TAGLN2 levels significantly increased in patients with cholangiocarcinoma. Compared to CA19-9, the only existing cancer marker, TAGLN2, showed a significantly higher ROC AUC in distinguishing patients with cholangiocarcinoma than those with normal or benign disease (TAGLN2 vs. CA19-9:0.901 vs. 0.799, *P* = 0.026), moreover, combining with CA19-9, ROC AUC was increased even 0.948. These results suggest that TAGLN2 can be used as a diagnostic marker for cholangiocarcinoma. In particular, 37 of the 44 CA19-9 negative patients (84.1%) showed TAGLN2 elevation above the cutoff, suggesting that TAGLN2 can overcome the low sensitivity of CA19-9. However, because of the unavailability of an efficient TAGLN2 ELISA kit at present, quantitative analysis of TAGLN2 in blood was performed using WB densitometry, which is difficult to apply consistently in various clinical situations. To use TAGLN2 in the blood for diagnosing cholangiocarcinoma, a technical method that can provide more robust and consistent results is needed.

In conclusion, the present study shows that *TAGLN2* plays a specific role in tumor proliferation, migration, and invasion and is involved in chemoresistance by inducing EMT-like changes. Targeting *TAGLN2* is expected to be a successful anti-cancer therapy for advanced cancer following chemotherapy failure. Further studies to clarify the signaling network and mechanisms of *TAGLN2* in carcinogenesis and drug resistance should be conducted to develop new therapeutic approaches for treating chemorefractory BTC.

### Electronic supplementary material

Below is the link to the electronic supplementary material.

Supplementary Material 1

Supplementary Material 2

Supplementary Material 3

## Data Availability

The datasets generated and/or analyzed during the current study are available in the NCBI Gene Expression Omnibus (GEO) repository [GSE233893].

## Abbreviations

BTC: Biliary tract cancer
CSC: Cancer stem cell
TAGLN2: Transgelin-2
EMT: Epithelial-mesenchymal transition
shRNA: Short hairpin RNAs
ROC: Receiver operating characteristic curve
AUC: Area under the ROC curve
CI: Combination index
IHCC: Intrahepatic cholangiocarcinoma
PHCC: Perihilar cholangiocarcinoma
CBD: Cancer, common bile duct cancer
CAFs: Cancer-associated fibroblasts
DFS: Disease free survival
OS: Overall survival
