# Supplementary material for: Transgelin-2, a novel cancer stem cell-related biomarker, is a diagnostic and therapeutic target for biliary tract cancer
Source: BMC Cancer. 2024 Mar 20;24:357. doi: 10.1186/s12885-024-12082-3 (PMC10953140; doi:10.1186/s12885-024-12082-3)
Supplement: Supplementary file 1 — Supplementary Material 1 [file 12885_2024_12082_MOESM1_ESM.pdf]

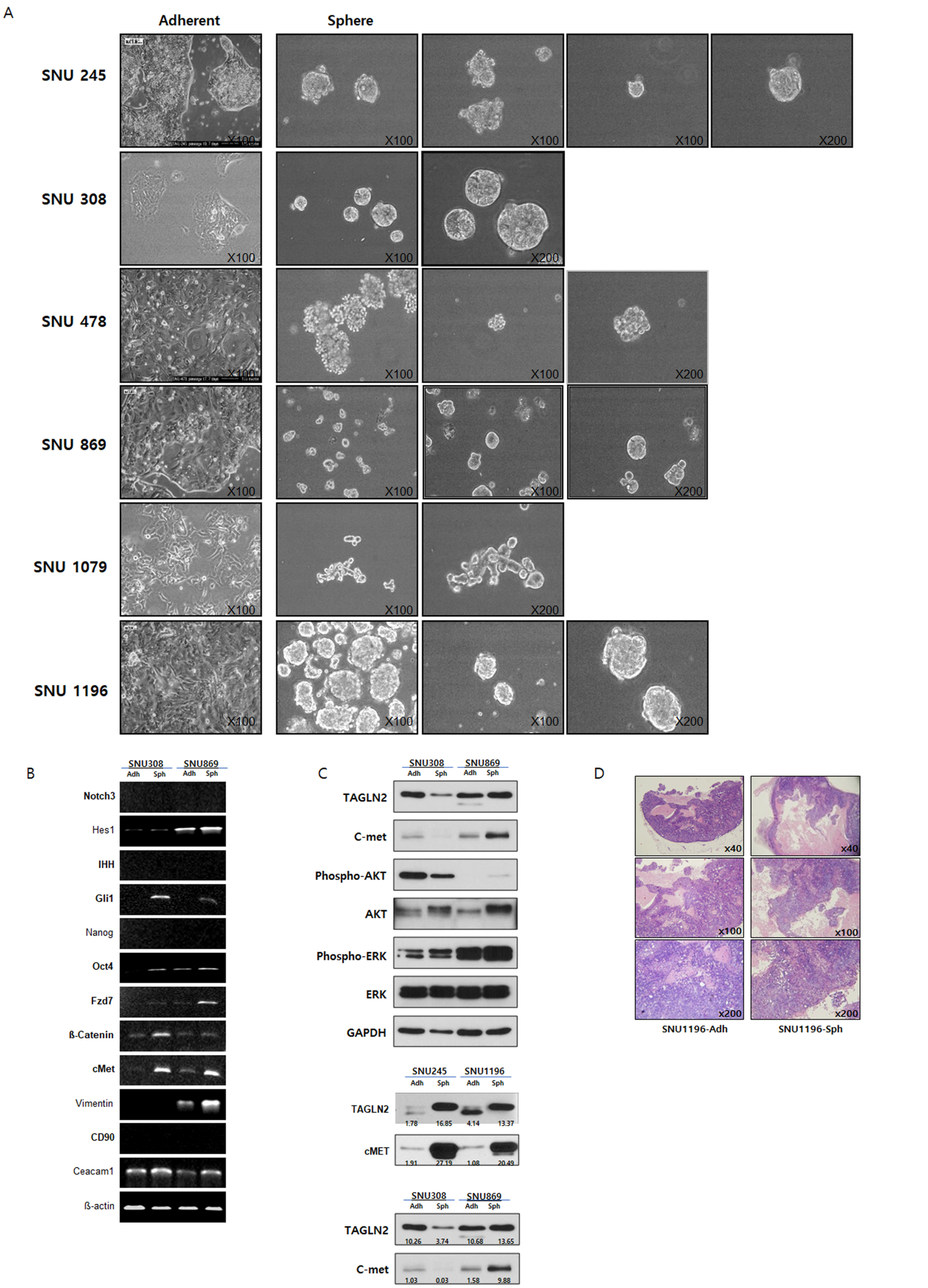


**Supplementary Fig. 1.** Expression of *Transgelin-2* (TAGLN2) in the biliary tract cancer cell (BTC)-spheres. (**A**) Sphere-formation assays of BTC cell lines, including SNU245, SNU308, SNU478, SNU869, SNU1079, and SNU1196; (100×, 200×). (**B**) Differentially expressed genes and (**C**) proteins analyzed in sphere-cultured cells in SNU308 and SNU869. (**D**) Tumorigenesis was performed using adherent and sphere-cultured SNU1196 *in vivo* (40×, 100×, 200×)

A

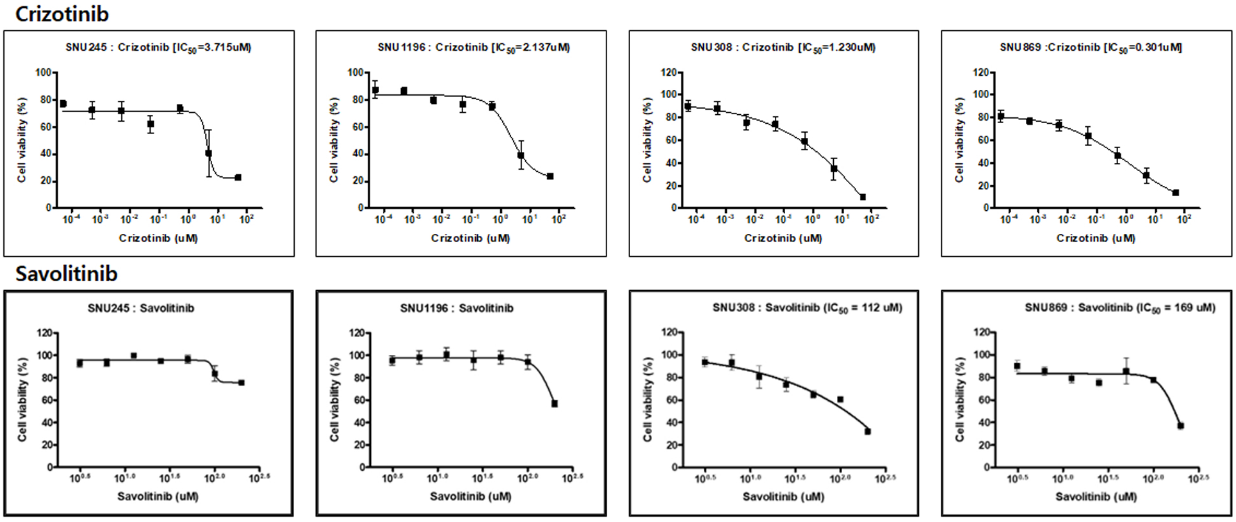


B

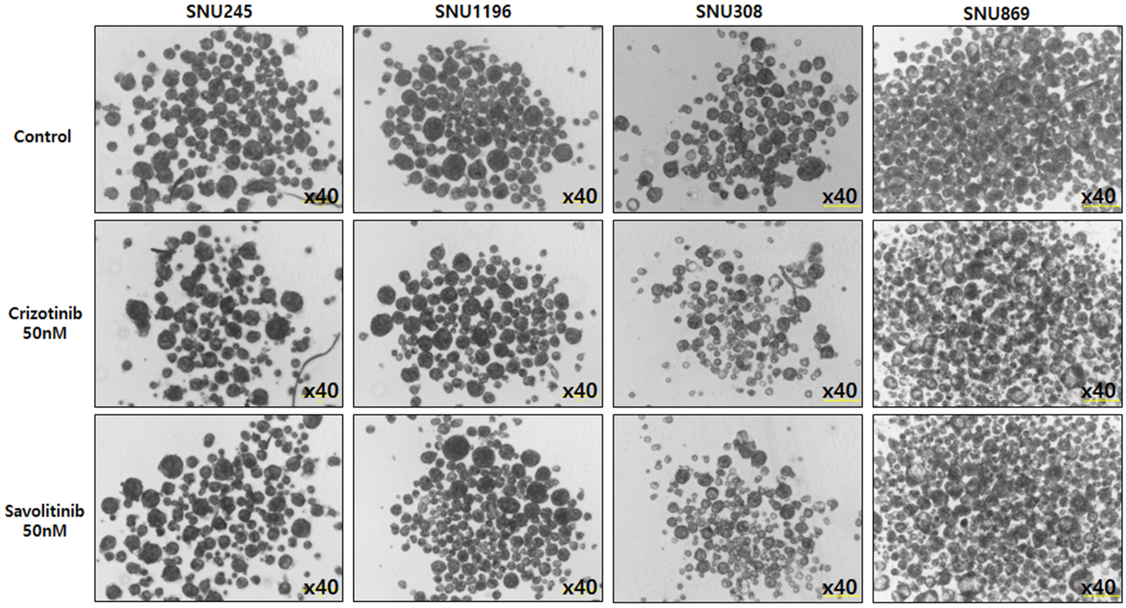


**Supplementary Fig. 2.** Sensitivity to cMET inhibitors. (**A, B**) Sensitivity of **(A)** cell lines and (**B**) spheres to crizotinib and savolitinib (at 50 nM each).

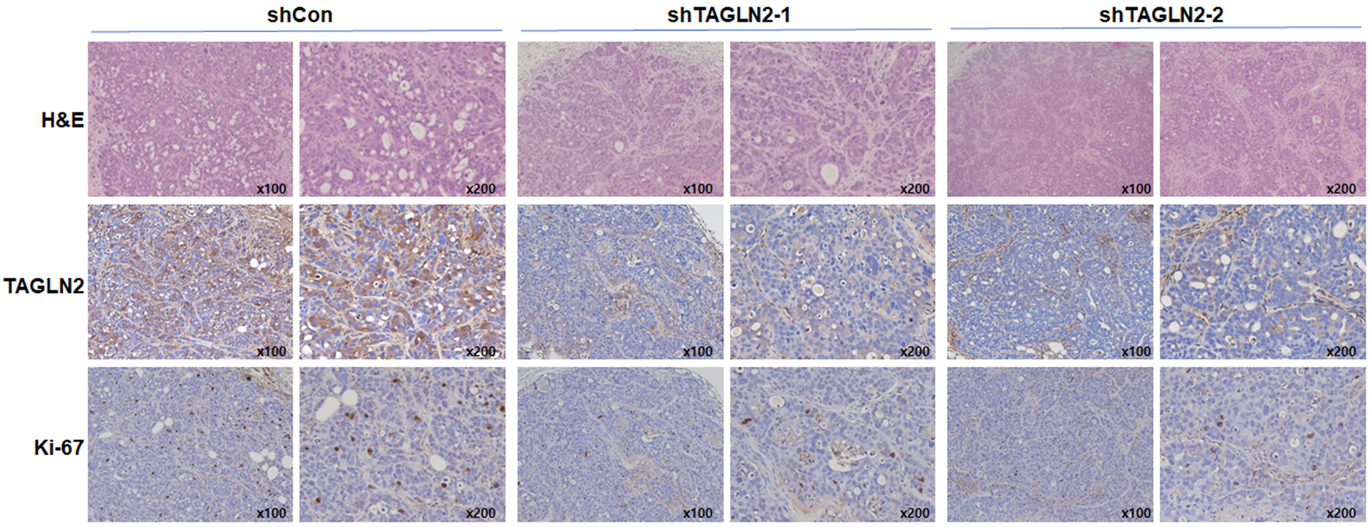


**Supplementary Fig. 3.** The expression of Ki67, a cell proliferation marker in *TAGLN2*-suppressed mice tumor tissues (100×, 200×).

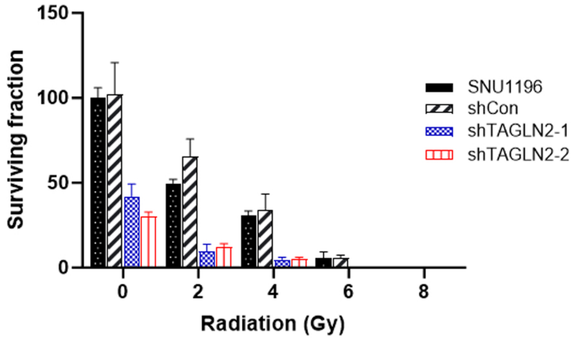


**Supplementary Fig. 4.** The surviving fractions at different doses in cells in which *TAGLN2* is suppressed using shRNA (shTAGLN2-1, -2) compared to control vector-transfected cells.

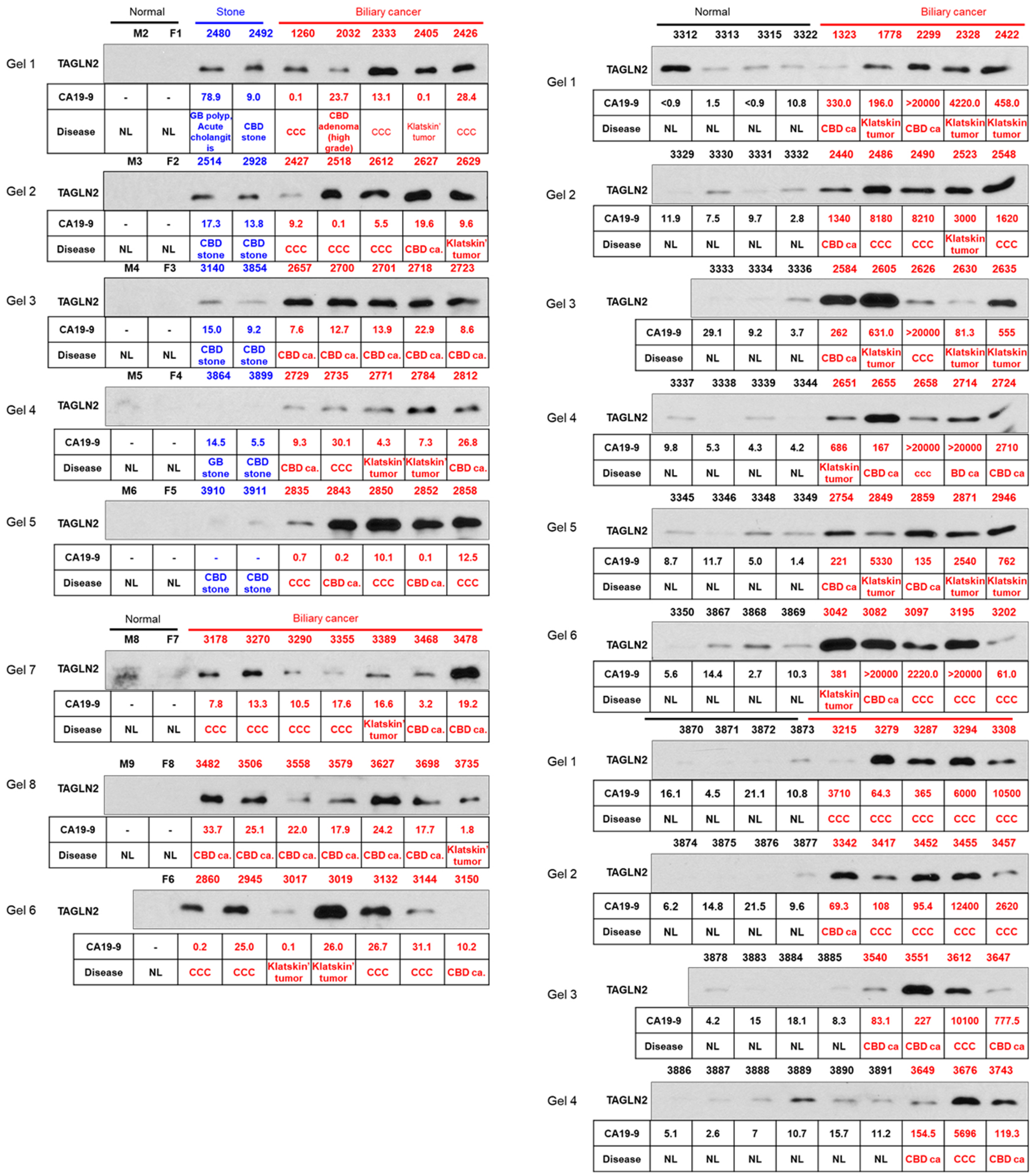

Normal / Stone / Biliary cancer

| Gel 1 | M2 | F1 | 2480 | 2492 | 1260 | 2032 | 2333 | 2405 | 2426 |
|---|---|---|---|---|---|---|---|---|---|
| CA19-9 | - | - | 78.9 | 9.0 | 0.1 | 23.7 | 13.1 | 0.1 | 28.4 |
| Disease | NL | NL | GB polyp, Acute cholangitis | CBD stone | CCC | CBD adenoma (high grade) | CCC | Klatskin' tumor | CCC |

| Gel 2 | M3 | F2 | 2514 | 2928 | 2427 | 2518 | 2612 | 2627 | 2629 |
|---|---|---|---|---|---|---|---|---|---|
| CA19-9 | - | - | 17.3 | 13.8 | 9.2 | 0.1 | 5.5 | 19.6 | 9.6 |
| Disease | NL | NL | CBD stone | CBD stone | CCC | CCC | CCC | CBD ca. | Klatskin' tumor |

| Gel 3 | M4 | F3 | 3140 | 3854 | 2657 | 2700 | 2701 | 2718 | 2723 |
|---|---|---|---|---|---|---|---|---|---|
| CA19-9 | - | - | 15.0 | 9.2 | 7.6 | 12.7 | 13.9 | 22.9 | 8.6 |
| Disease | NL | NL | CBD stone | CBD stone | CBD ca. | CBD ca. | CBD ca. | CBD ca. | CBD ca. |

| Gel 4 | M5 | F4 | 3864 | 3899 | 2729 | 2735 | 2771 | 2784 | 2812 |
|---|---|---|---|---|---|---|---|---|---|
| CA19-9 | - | - | 14.5 | 5.5 | 9.3 | 30.1 | 4.3 | 7.3 | 26.8 |
| Disease | NL | NL | GB stone | CBD stone | CBD ca. | CCC | Klatskin' tumor | Klatskin' tumor | CBD ca. |

| Gel 5 | M6 | F5 | 3910 | 3911 | 2835 | 2843 | 2850 | 2852 | 2858 |
|---|---|---|---|---|---|---|---|---|---|
| CA19-9 | - | - | - | - | 0.7 | 0.2 | 10.1 | 0.1 | 12.5 |
| Disease | NL | NL | CBD stone | CBD stone | CCC | CBD ca. | CCC | CBD ca. | CCC |

Normal / Biliary cancer

| Gel 7 | M8 | F7 | 3178 | 3270 | 3290 | 3355 | 3389 | 3468 | 3478 |
|---|---|---|---|---|---|---|---|---|---|
| CA19-9 | - | - | 7.8 | 13.3 | 10.5 | 17.6 | 16.6 | 3.2 | 19.2 |
| Disease | NL | NL | CCC | CCC | CCC | CCC | Klatskin' tumor | CBD ca. | CBD ca. |

| Gel 8 | M9 | F8 | 3482 | 3506 | 3558 | 3579 | 3627 | 3698 | 3735 |
|---|---|---|---|---|---|---|---|---|---|
| CA19-9 | - | - | 33.7 | 25.1 | 22.0 | 17.9 | 24.2 | 17.7 | 1.8 |
| Disease | NL | NL | CBD ca. | CBD ca. | CBD ca. | CBD ca. | CBD ca. | CBD ca. | Klatskin' tumor |

| Gel 6 | F6 | 2860 | 2945 | 3017 | 3019 | 3132 | 3144 | 3150 |
|---|---|---|---|---|---|---|---|---|
| CA19-9 | - | 0.2 | 25.0 | 0.1 | 26.0 | 26.7 | 31.1 | 10.2 |
| Disease | NL | CCC | CCC | Klatskin' tumor | Klatskin' tumor | CCC | CCC | CBD ca. |

Normal / Biliary cancer

| Gel 1 | 3312 | 3313 | 3315 | 3322 | 1323 | 1778 | 2299 | 2328 | 2422 |
|---|---|---|---|---|---|---|---|---|---|
| CA19-9 | <0.9 | 1.5 | <0.9 | 10.8 | 330.0 | 196.0 | >20000 | 4220.0 | 458.0 |
| Disease | NL | NL | NL | NL | CBD ca | Klatskin tumor | CBD ca | Klatskin tumor | Klatskin tumor |

| Gel 2 | 3329 | 3330 | 3331 | 3332 | 2440 | 2486 | 2490 | 2523 | 2548 |
|---|---|---|---|---|---|---|---|---|---|
| CA19-9 | 11.9 | 7.5 | 9.7 | 2.8 | 1340 | 8180 | 8210 | 3000 | 1620 |
| Disease | NL | NL | NL | NL | CBD ca | CCC | CCC | Klatskin tumor | CCC |

| Gel 3 | 3333 | 3334 | 3336 | 2584 | 2605 | 2626 | 2630 | 2635 |
|---|---|---|---|---|---|---|---|---|
| CA19-9 | 29.1 | 9.2 | 3.7 | 262 | 631.0 | >20000 | 81.3 | 555 |
| Disease | NL | NL | NL | CBD ca | Klatskin tumor | CCC | Klatskin tumor | Klatskin tumor |

| Gel 4 | 3337 | 3338 | 3339 | 3344 | 2651 | 2655 | 2658 | 2714 | 2724 |
|---|---|---|---|---|---|---|---|---|---|
| CA19-9 | 9.8 | 5.3 | 4.3 | 4.2 | 686 | 167 | >20000 | >20000 | 2710 |
| Disease | NL | NL | NL | NL | Klatskin tumor | CBD ca | ccc | BD ca | CBD ca |

| Gel 5 | 3345 | 3346 | 3348 | 3349 | 2754 | 2849 | 2859 | 2871 | 2946 |
|---|---|---|---|---|---|---|---|---|---|
| CA19-9 | 8.7 | 11.7 | 5.0 | 1.4 | 221 | 5330 | 135 | 2540 | 762 |
| Disease | NL | NL | NL | NL | CBD ca | Klatskin tumor | CBD ca | Klatskin tumor | Klatskin tumor |

| Gel 6 | 3350 | 3867 | 3868 | 3869 | 3042 | 3082 | 3097 | 3195 | 3202 |
|---|---|---|---|---|---|---|---|---|---|
| CA19-9 | 5.6 | 14.4 | 2.7 | 10.3 | 381 | >20000 | 2220.0 | >20000 | 61.0 |
| Disease | NL | NL | NL | NL | Klatskin tumor | CBD ca | CCC | CCC | CCC |

| Gel 1 | 3870 | 3871 | 3872 | 3873 | 3215 | 3279 | 3287 | 3294 | 3308 |
|---|---|---|---|---|---|---|---|---|---|
| CA19-9 | 16.1 | 4.5 | 21.1 | 10.8 | 3710 | 64.3 | 365 | 6000 | 10500 |
| Disease | NL | NL | NL | NL | CCC | CCC | CCC | CCC | CCC |

| Gel 2 | 3874 | 3875 | 3876 | 3877 | 3342 | 3417 | 3452 | 3455 | 3457 |
|---|---|---|---|---|---|---|---|---|---|
| CA19-9 | 6.2 | 14.8 | 21.5 | 9.6 | 69.3 | 108 | 95.4 | 12400 | 2620 |
| Disease | NL | NL | NL | NL | CBD ca | CCC | CCC | CCC | CCC |

| Gel 3 | 3878 | 3883 | 3884 | 3885 | 3540 | 3551 | 3612 | 3647 |
|---|---|---|---|---|---|---|---|---|
| CA19-9 | 4.2 | 15 | 18.1 | 8.3 | 83.1 | 227 | 10100 | 777.5 |
| Disease | NL | NL | NL | NL | CBD ca | CBD ca | CCC | CBD ca |

| Gel 4 | 3886 | 3887 | 3888 | 3889 | 3890 | 3891 | 3649 | 3676 | 3743 |
|---|---|---|---|---|---|---|---|---|---|
| CA19-9 | 5.1 | 2.6 | 7 | 10.7 | 15.7 | 11.2 | 154.5 | 5696 | 119.3 |
| Disease | NL | NL | NL | NL | NL | NL | CBD ca | CCC | CBD ca |

**Supplementary Fig. 5.** TAGLN2 expression in patient serum samples.

A

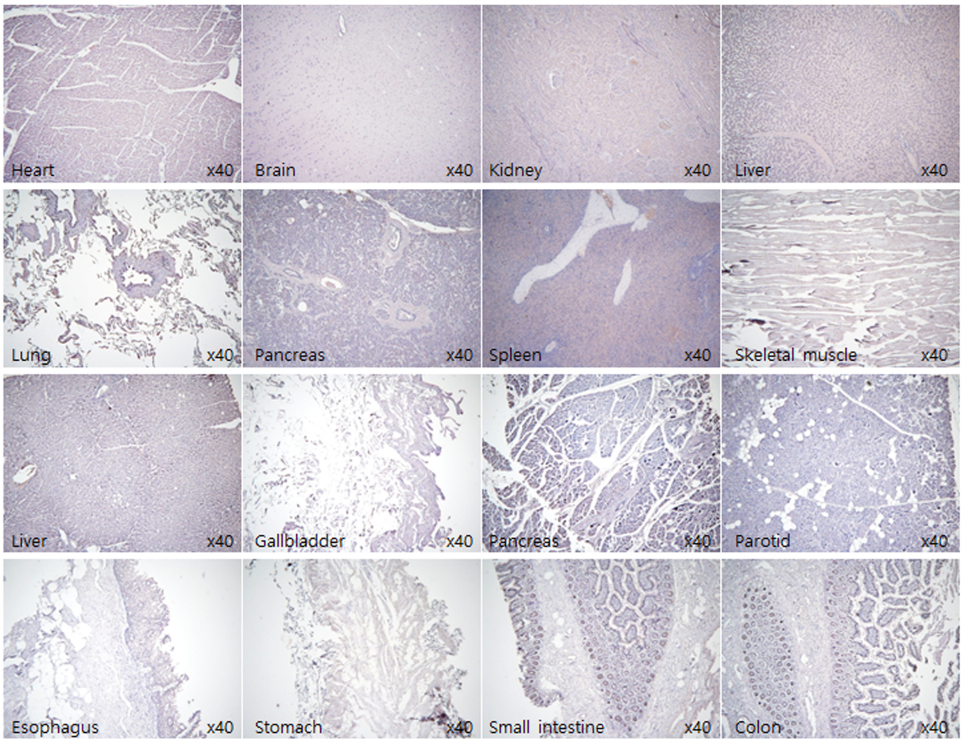


B

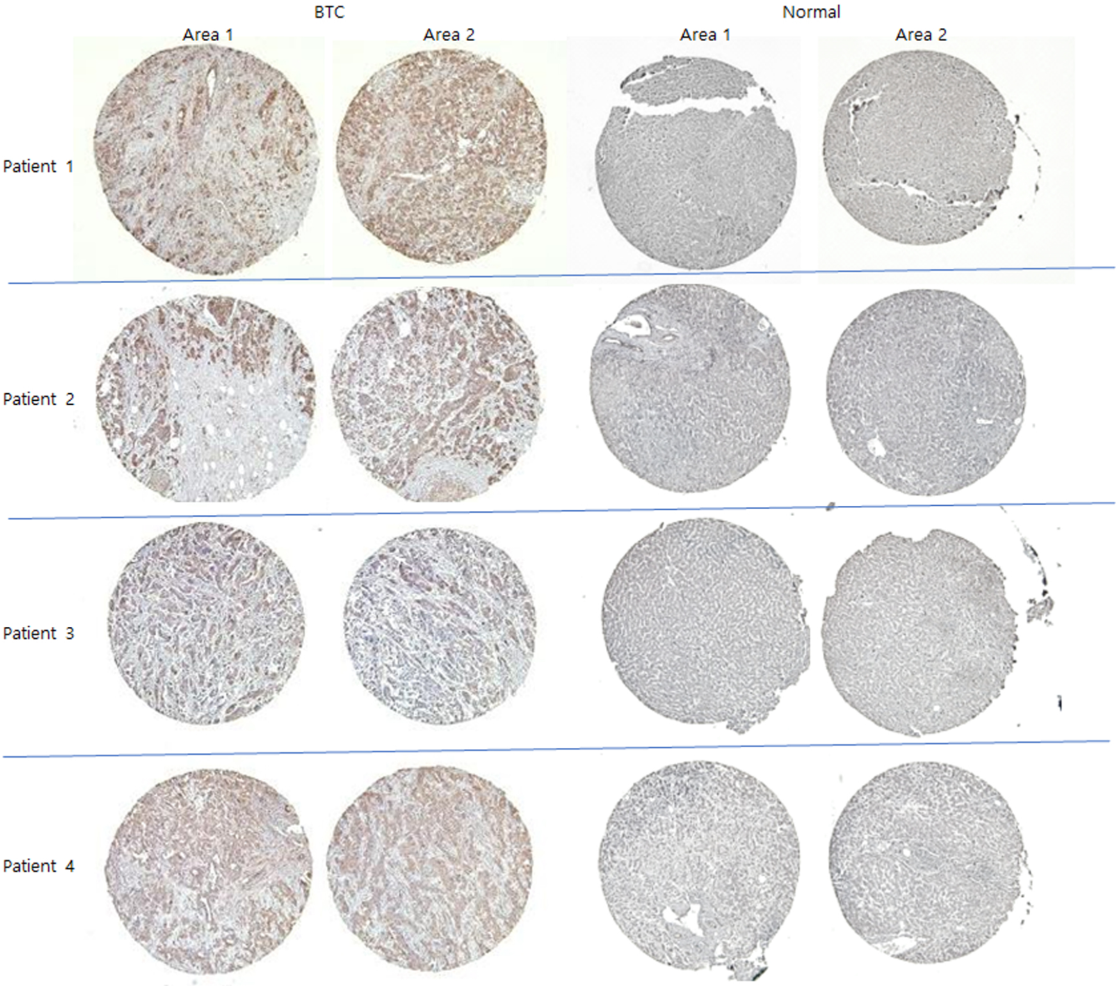


**Supplementary Fig. 6.** TAGLN2 expression in normal and patient tissues. (**A**) TAGLN2 expression in normal human tissues assessed using IHC (40×). (**B**) Representative images show the expression of TAGLN2 in cancer and adjacent normal BTC surgical tissues. IHC, Immunohistochemistry.

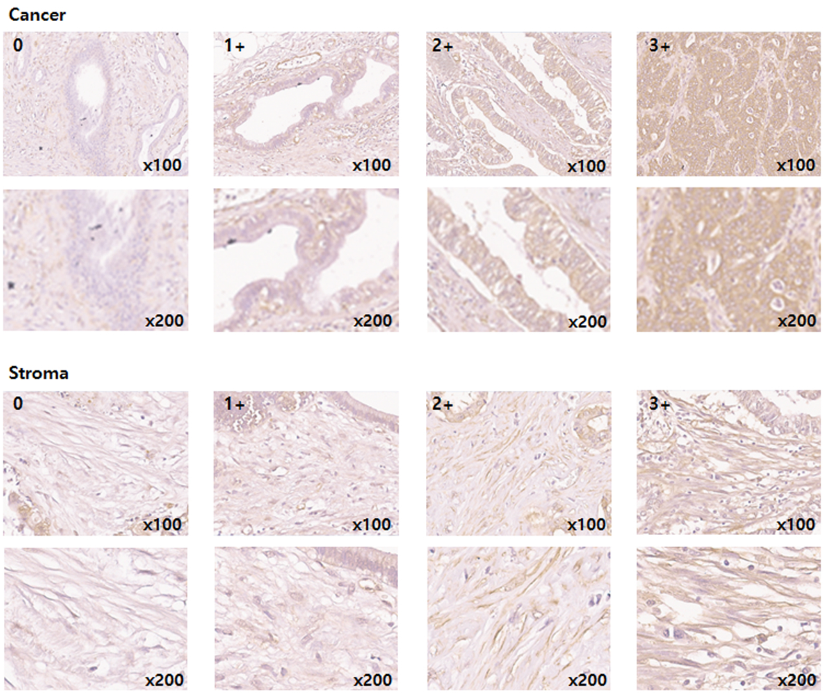


**Supplementary Fig. 7.** TAGLN2 expression in patient cancer tissues (100×, 200×)

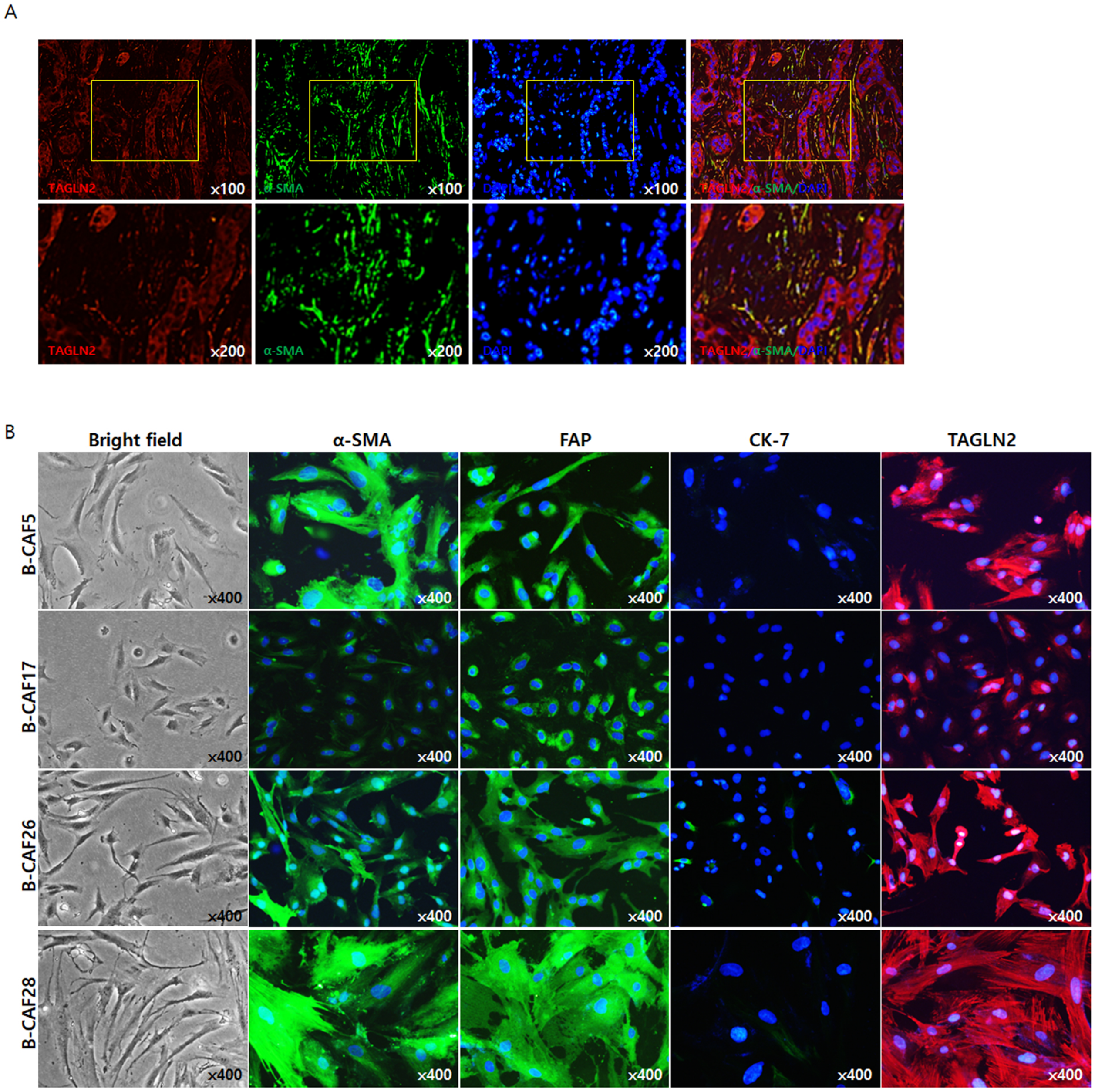


**Supplementary Fig. 8.** TAGLN2 expression in patient tissues and cancer-associated fibroblasts. (**A**) IF findings in BTC tissue show overexpression of TAGLN2 in the stroma of BTC tissue, and its location coincided with α-SMA expression (100×, 200×) (**B**) TAGLN2 expression in the CAFs from BTC assessed using IF (400×); α-SMA, alpha-smooth muscle actin; IF, Immunofluorescence.
