# Supplementary material for: Transgelin-2, a novel cancer stem cell-related biomarker, is a diagnostic and therapeutic target for biliary tract cancer
Source: BMC Cancer. 2024 Mar 20;24:357. doi: 10.1186/s12885-024-12082-3 (PMC10953140; doi:10.1186/s12885-024-12082-3)
Supplement: Supplementary file 2 — Supplementary Material 2 [file 12885_2024_12082_MOESM2_ESM.docx]

**Table S1**. Combination Index

**Table S2. Baseline characteristics of patients with TAGLN2 western blot**

|  |  | **Normal controls (n=40)** | | **Biliary stones (n=10)** | | **Biliary cancers (n=89)** | |
| --- | --- | --- | --- | --- | --- | --- | --- |
| **AGE (SD)** |  | 45.5 | (9.2) | 59.1 | (12.1) | 63.2 | (10.5) |
| **Sex (%)** | Female | 12 | (30.0%) | 4 | (40.0%) | 34 | (38.2%) |
|  | Male | 28 | (70.0%) | 6 | (60.0%) | 55 | (61.8%) |
| **Initial CA19-9, U/mL, (SD)** |  | 9 (6) | | 12 (7) | | 2417 (5377) | |
| **TAGLN2 expression, unit, (SD)** |  | 539 (776) | | 953 (1087) | | 3710 (2568) | |
| **Primary location (%)** | Intrahepatic |  |  |  |  | 29 | (32.6%) |
|  | Perihilar |  |  |  |  | 28 | (31.5%) |
|  | Distal CBD |  |  |  |  | 32 | (36.0%) |
| **Initial stage (%)** | I |  |  |  |  | 6 | (6.7%) |
|  | II |  |  |  |  | 35 | (39.3%) |
|  | III |  |  |  |  | 29 | (32.6%) |
|  | IV |  |  |  |  | 19 | (21.3%) |

**Table S3. Baseline characteristics according to cancer IHC index of TAGLN2**

|  |  | **Low Cancer IHC Index (N=10)** | | **High Cancer IHC index (N=31)** | | **P value** |
| --- | --- | --- | --- | --- | --- | --- |
| **AGE (SD)** |  | 60.3 | (8.4) | 65.2 | (11.9) | 0.239 |
| **Sex (%)** | Female | 6 | (60%) | 16 | (52%) | 0.644 |
|  | Male | 4 | (40%) | 15 | (48%) |  |
| **HTN (%)** | Yes | 8 | (80%) | 21 | (68%) | 0.459 |
|  | No | 2 | (20%) | 10 | (32%) |  |
| **DM (%)** | Yes | 6 | (60%) | 15 | (48%) | 0.523 |
|  | No | 4 | (40%) | 16 | (52%) |  |
| **Primary location (%)** | Intrahepatic | 1 | (10%) | 7 | (23%) | 0.743 |
|  | Perihilar | 3 | (30%) | 6 | (19%) |  |
|  | Distal CBD | 4 | (40%) | 10 | (32%) |  |
|  | GB | 2 | (20%) | 8 | (26%) |  |
| **Initial CA19-9, U/mL, (SD)** |  | 2682 | (6265) | 1463 | (4959) | 0.530 |
| **Initial stage (%)** | I | 1 | (10%) | 5 | (32%) | 0.154 |
|  | II | 5 | (50%) | 9 | (26%) |  |
|  | III | 4 | (40%) | 7 | (26%) |  |
|  | IV | 0 | (0%) | 10 | (26%) |  |
| **Adjuvant therapy (%)** | No | 3 | (30%) | 13 | (26%) | 0.501 |
|  | Yes | 7 | (70%) | 18 | (26%) |  |
| **Recurrence** | No | 10 | (100%) | 28 | (26%) | 0.307 |
|  | Yes | 0 | (0%) | 3 | (26%) |  |

**Table S4. Baseline characteristics according to stroma IHC index of TAGLN2**

|  |  | **Low Stroma IHC Index (N=19)** | | **High Stroma IHC index (N=22)** | | **P value** |
| --- | --- | --- | --- | --- | --- | --- |
| **AGE (SD)** |  | 64.9 | (10.5) | 63.1 | (12.1) | 0.621 |
| **Sex (%)** | Female | 11 | (58%) | 11 | (50%) | 0.613 |
|  | Male | 8 | (42%) | 11 | (50%) |  |
| **HTN (%)** | Yes | 12 | (63%) | 17 | (77%) | 0.322 |
|  | No | 7 | (37%) | 5 | (23%) |  |
| **DM (%)** | Yes | 12 | (63%) | 9 | (41%) | 0.155 |
|  | No | 7 | (37%) | 13 | (59%) |  |
| **Primary location (%)** | Intrahepatic | 2 | (11%) | 6 | (27%) | 0.221 |
|  | Perihilar | 3 | (16%) | 6 | (27%) |  |
|  | Distal CBD | 7 | (37%) | 7 | (32%) |  |
|  | GB | 7 | (37%) | 3 | (14%) |  |
| **Initial CA19-9, U/mL, (SD)** |  | 2347 | (6235) | 1255 | (4312) | 0.513 |
| **Initial stage (%)** | I | 2 | (11%) | 4 | (18%) | 0.910 |
|  | II | 7 | (37%) | 7 | (32%) |  |
|  | III | 5 | (26%) | 6 | (27%) |  |
|  | IV | 5 | (26%) | 5 | (23%) |  |
| **Adjuvant therapy (%)** | No | 8 | (42%) | 8 | (36%) | 0.707 |
|  | Yes | 11 | (58%) | 14 | (64%) |  |
| **Recurrence** | No | 17 | (89%) | 21 | (95%) | 0.463 |
|  | Yes | 2 | (11%) | 1 | (5%) |  |
