# Supplementary material for: Transgelin-2, a novel cancer stem cell-related biomarker, is a diagnostic and therapeutic target for biliary tract cancer
Source: BMC Cancer. 2024 Mar 20;24:357. doi: 10.1186/s12885-024-12082-3 (PMC10953140; doi:10.1186/s12885-024-12082-3)

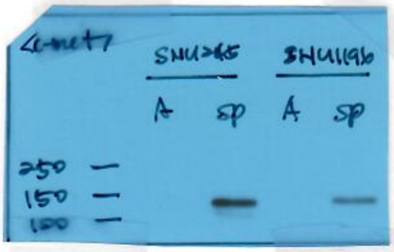
<c-met>
SNU245
SNU1196
A sp A sp
250
150
100

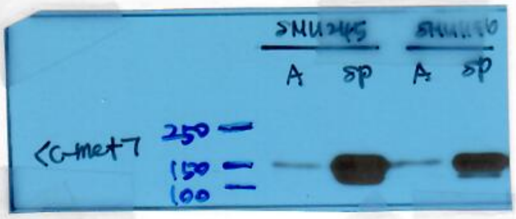
SNU245
SNU1196
A sp A sp
<c-met>
250
150
100

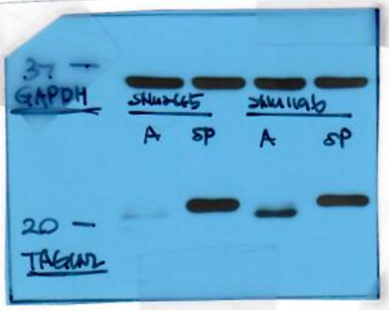
37
GAPDH
SNU245
SNU1196
A sp A sp
20
TAGLN2

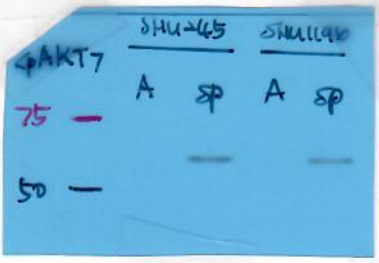
<pAKT>
SNU245
SNU1196
A sp A sp
75
50

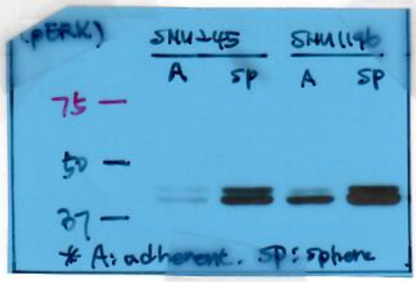
<pERK>
SNU245
SNU1196
A sp A sp
75
50
37
* A: adherent. SP: sphere

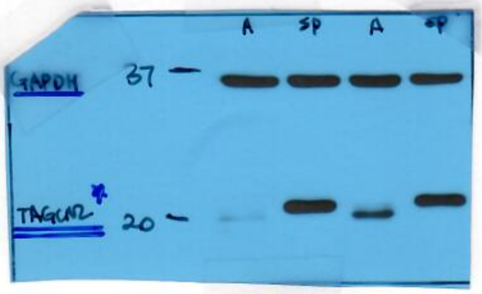
A sp A sp
GAPDH
37
TAGLN2*
20

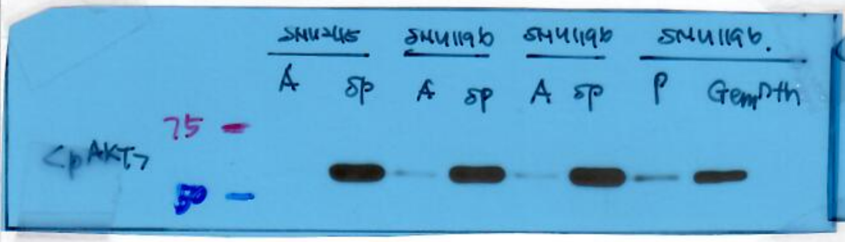
SNU245
SNU1196
SNU1196
SNU1196.
A sp A sp A sp P Gem7th
75
<pAKT>
50

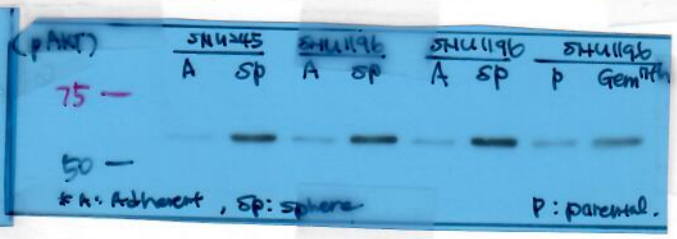
<pAKT>
SNU245
SNU1196
SNU1196
SNU1196
A sp A sp A sp P Gem7th
75
50
* A: Adherent, Sp: sphere
P: parental.

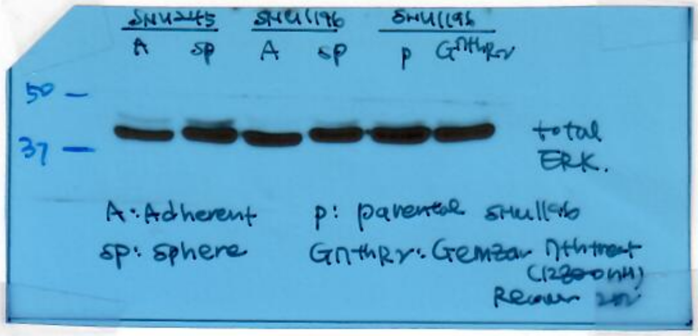
SNU245
SNU1196
SNU1196
A sp A sp p G7thRv
50
37
total ERK.
A: Adherent
sp: sphere
p: parental SNU1196
G7thRv: Gemzar 7thtreat (12800nM) Recover 2nd

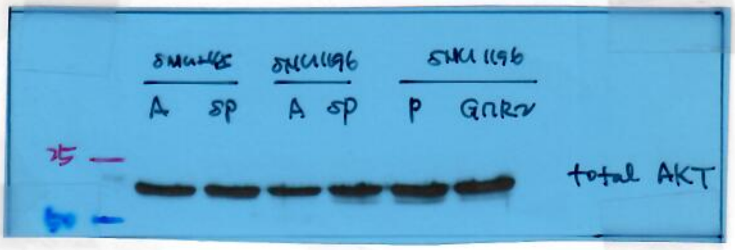
SNU245
SNU1196
SNU1196
A sp A sp P G7Rv
75
50
total AKT

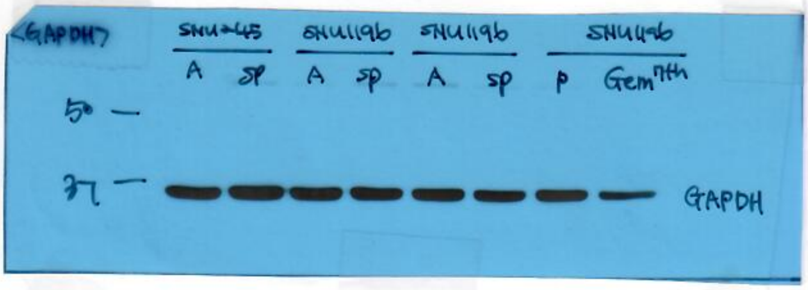
<GAPDH>
SNU245
SNU1196
SNU1196
SNU1196
A sp A sp A sp P Gem7th
50
37
GAPDH

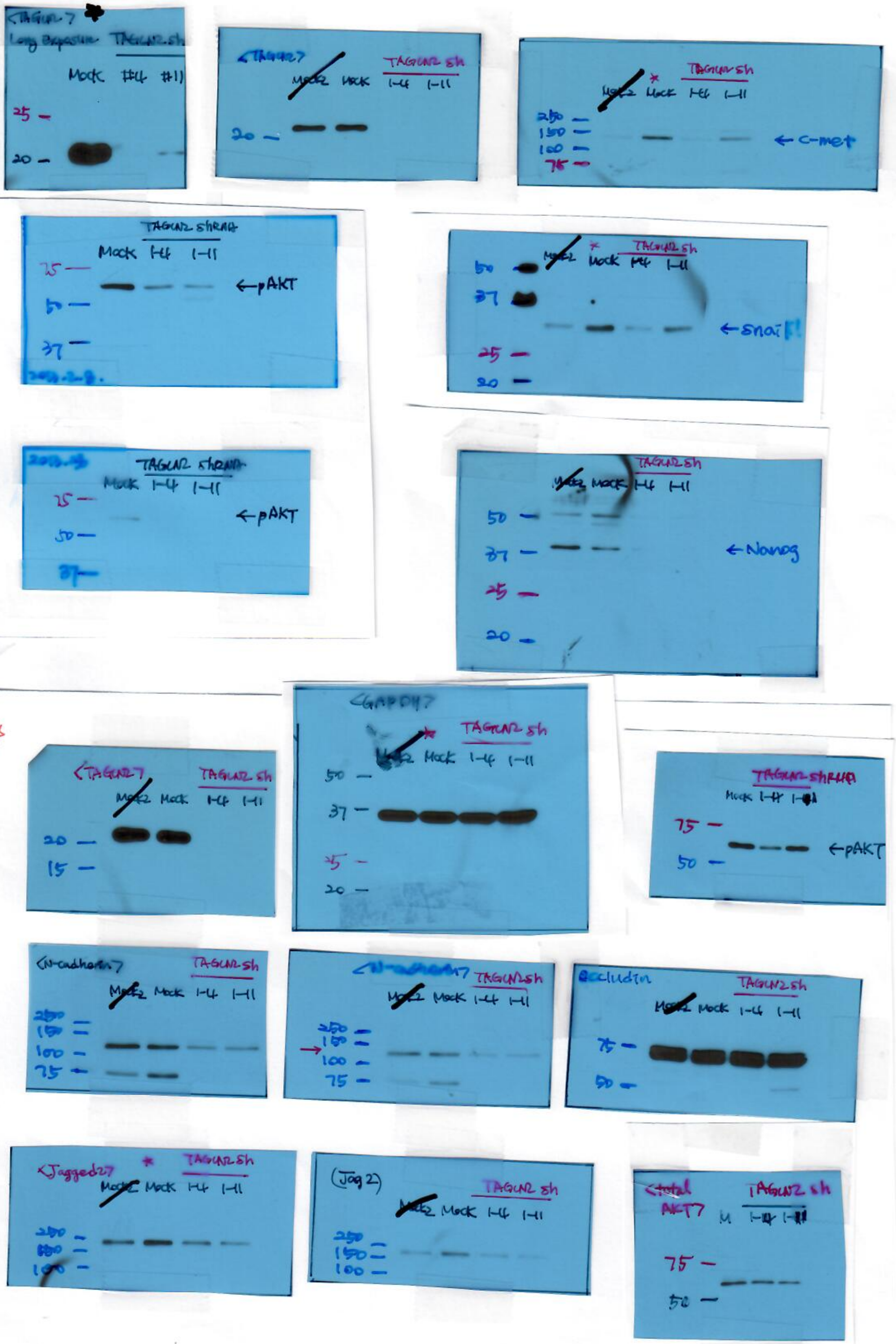

<TAGLN2>
Long Exposure
TAGLN2 sh
Mock #4 #11
25
20
<TAGLN2>
TAGLN2 sh
Mock2 Mock 1-4 1-11
20
TAGLN2 sh
Mock2 Mock 1-4 1-11
250
150
100
75
← c-met
TAGLN2 shRNA
Mock 1-4 1-11
75
50
37
← pAKT
TAGLN2 sh
Mock2 Mock 1-4 1-11
50
37
25
20
← snail
TAGLN2 shRNA
Mock 1-4 1-11
75
50
37
← pAKT
TAGLN2 sh
Mock2 Mock 1-4 1-11
50
37
25
20
← Nanog
<TAGLN2>
TAGLN2 sh
Mock2 Mock 1-4 1-11
20
15
<GAPDH>
TAGLN2 sh
Mock2 Mock 1-4 1-11
50
37
25
20
TAGLN2 shRNA
Mock 1-4 1-11
75
50
← pAKT
<N-cadherin>
TAGLN2 sh
Mock2 Mock 1-4 1-11
250
150
100
75
TAGLN2 sh
Mock2 Mock 1-4 1-11
250
150
100
75
Occludin
TAGLN2 sh
Mock2 Mock 1-4 1-11
75
50
<Jagged2>
TAGLN2 sh
Mock2 Mock 1-4 1-11
250
150
100
(Jag2)
TAGLN2 sh
Mock2 Mock 1-4 1-11
250
150
100
<total AKT>
TAGLN2 sh
M 1-4 1-11
75
50

※ SNU308 cell line.

* TAGLN2 siRNA 33nM. 72hr. transfection.
* protein 20μg loading

(2번 실험)

P : parental SNU308

C : control siRNA

T : TAGLN2 siRNA

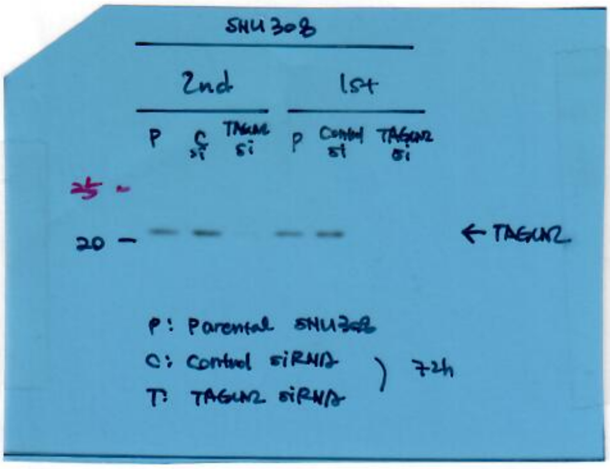

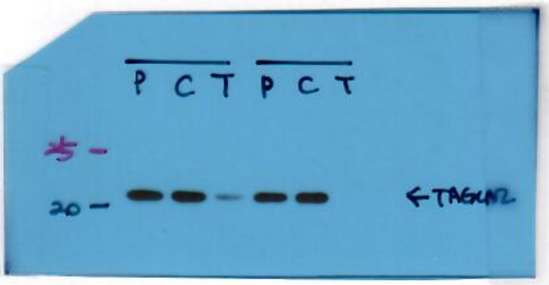

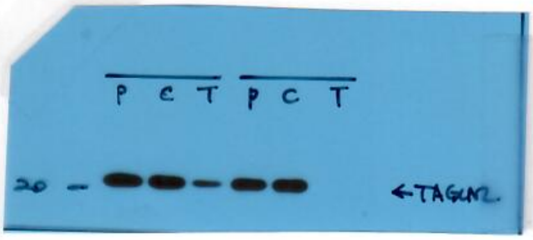

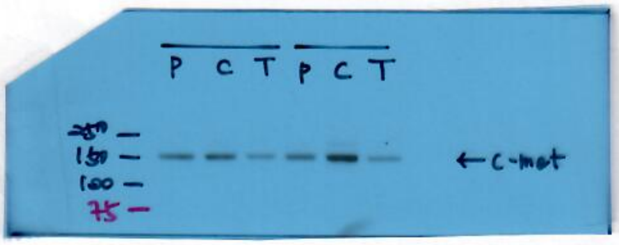

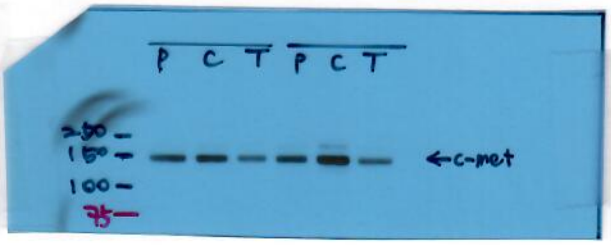

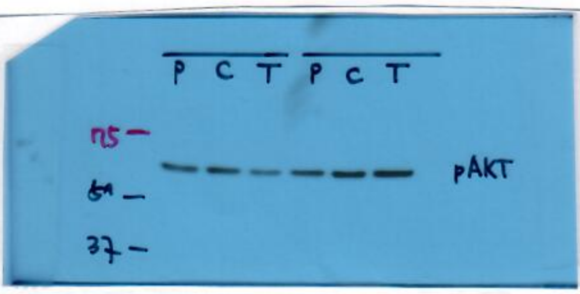

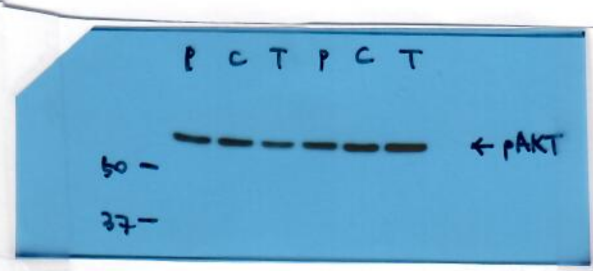

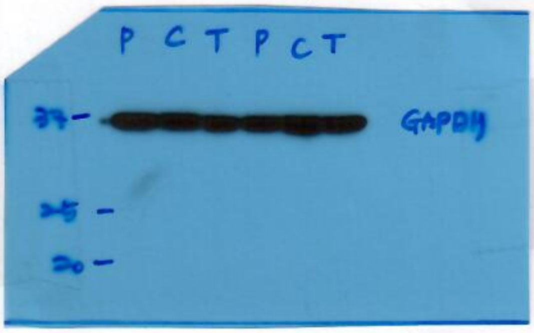

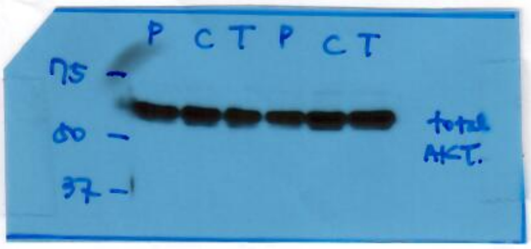

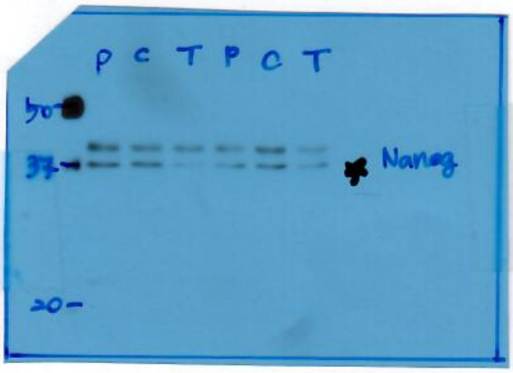

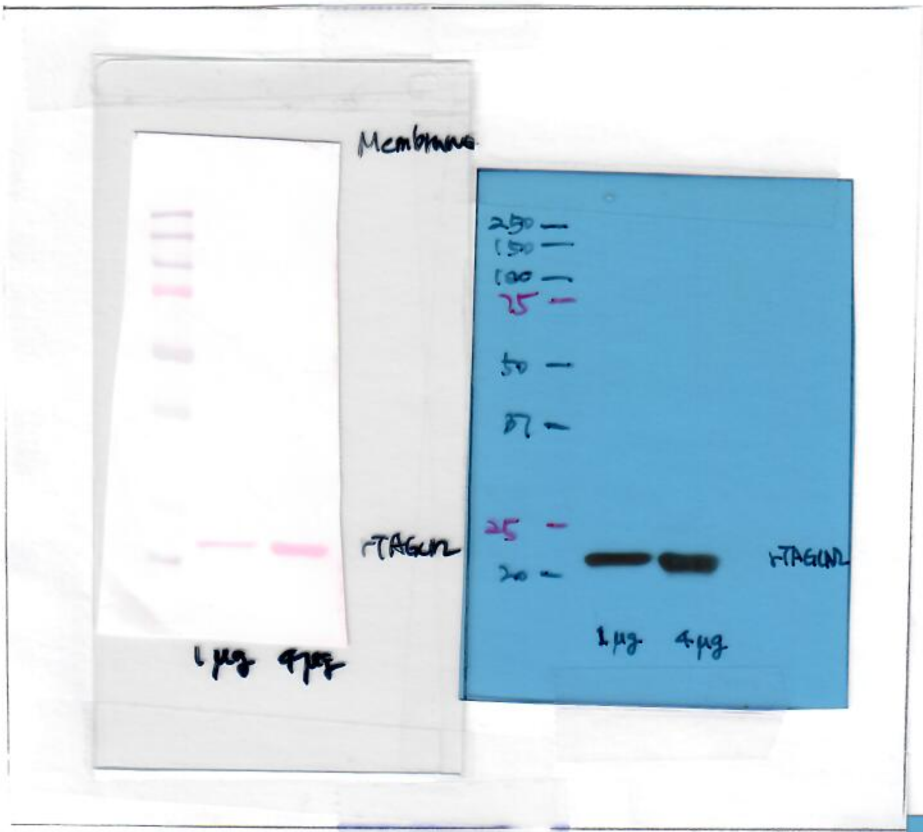
Membrane
rTAGLN2
1μg 4μg
250
150
100
75
50
37
25
20
rTAGLN2
1μg 4μg

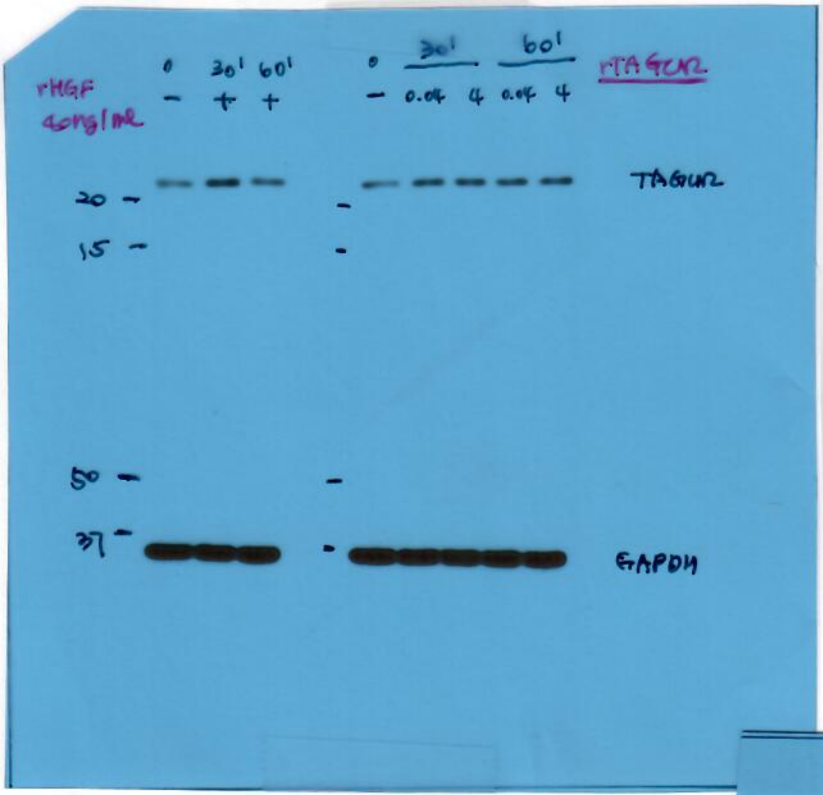
rHGF
40ng/ml
0 30' 60'
− + +
0 30' 60'
− 0.04 4 0.04 4
rTAGLN2
TAGLN2
20
15
50
37
GAPDH

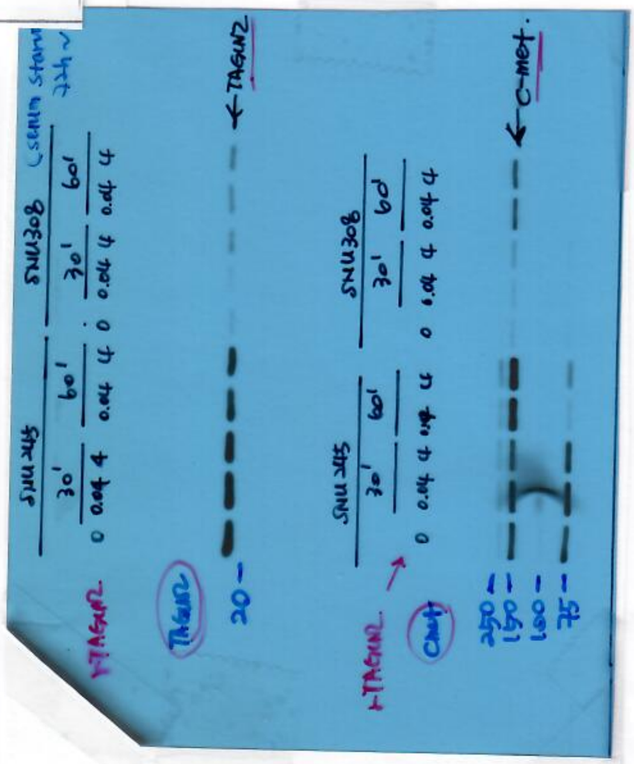
SNU245
SNU308
30' 60'
0 0.04 4 0.04 4
rTAGLN2
TAGLN2
20
← TAGLN2
cMet
250
150
100
75
← c-met

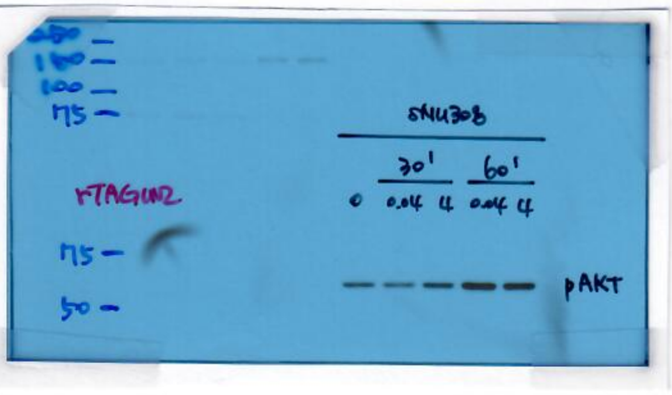
250
150
100
75
SNU308
30' 60'
0 0.04 4 0.04 4
rTAGLN2
75
50
pAKT

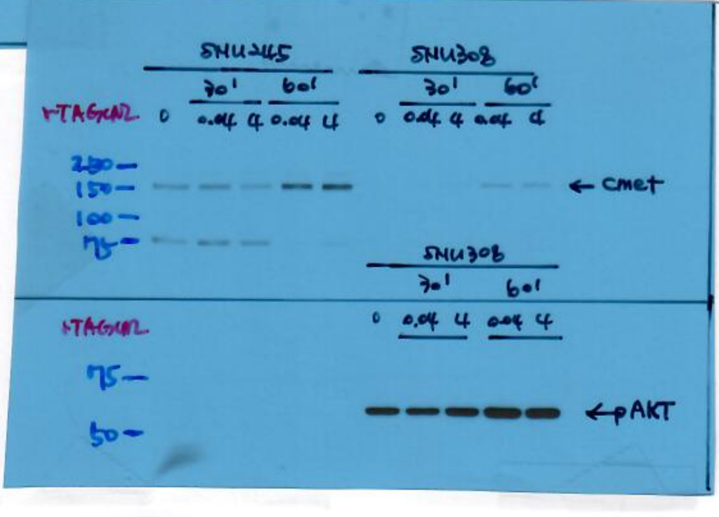
SNU245
SNU308
30' 60'
rTAGLN2 0 0.04 4 0.04 4
250
150
100
75
← cmet
SNU308
30' 60'
0 0.04 4 0.04 4
rTAGLN2
75
50
← pAKT

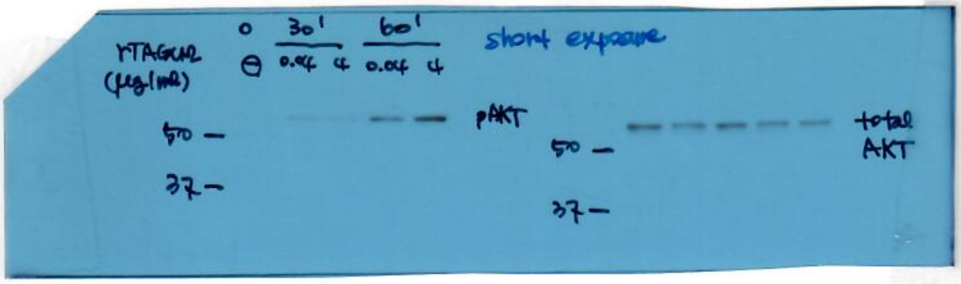
short exposure
rTAGLN2 (μg/ml)
0
30'
60'
0.04 4 0.04 4
pAKT
total AKT
50
37

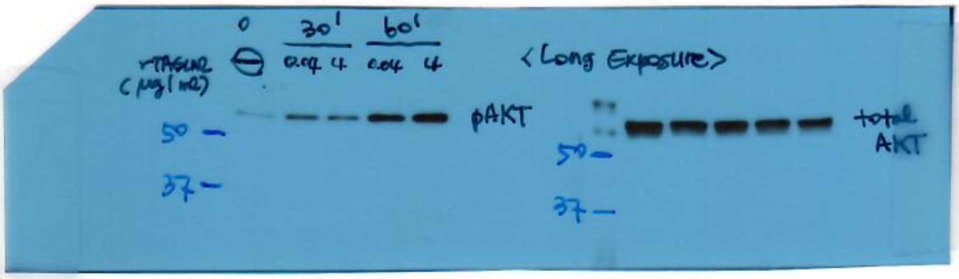
<Long Exposure>
rTAGLN2 (μg/ml)
0
30'
60'
0.04 4 0.04 4
pAKT
total AKT
50
37

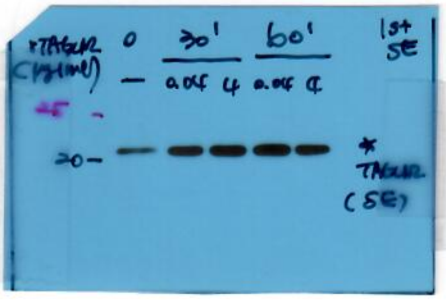
1st SE
rTAGLN2 (μg/ml)
0 30' 60'
0.04 4 0.04 4
20
* TAGLN2 (SE)

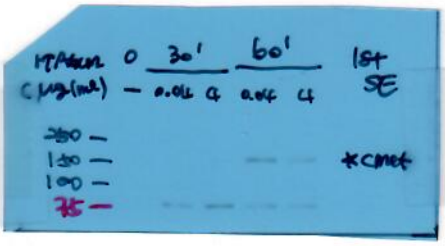
1st SE
rTAGLN2 (μg/ml)
0 30' 60'
0.04 4 0.04 4
250
150
100
75
*cmet

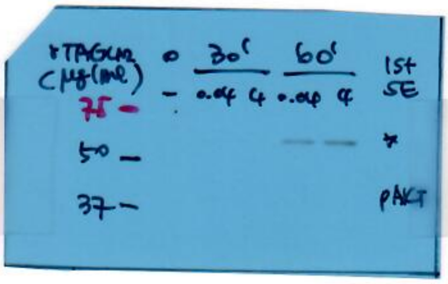
1st SE
rTAGLN2 (μg/ml)
0 30' 60'
0.04 4 0.04 4
75
50
37
*
pAKT

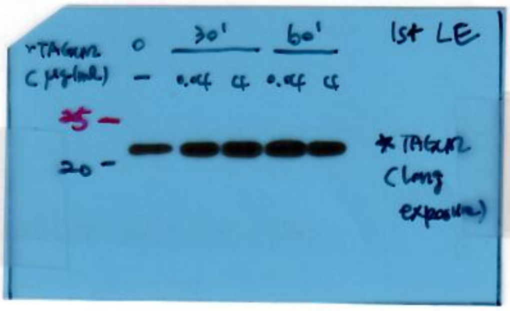
1st LE
rTAGLN2 (μg/ml)
0 30' 60'
0.04 4 0.04 4
75
20
* TAGLN2 (long exposure)

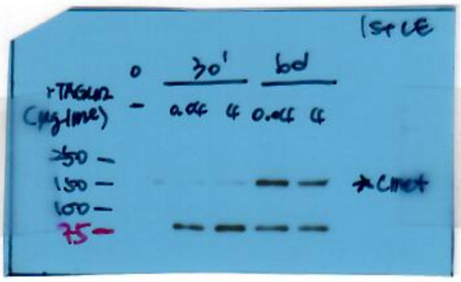
1st LE
rTAGLN2 (μg/ml)
0 30' 60'
0.04 4 0.04 4
250
150
100
75
*cmet

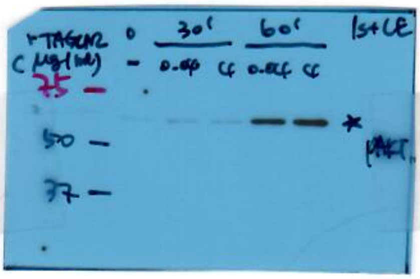
1st LE
rTAGLN2 (μg/ml)
0 30' 60'
0.04 4 0.04 4
75
50
37
*
pAKT

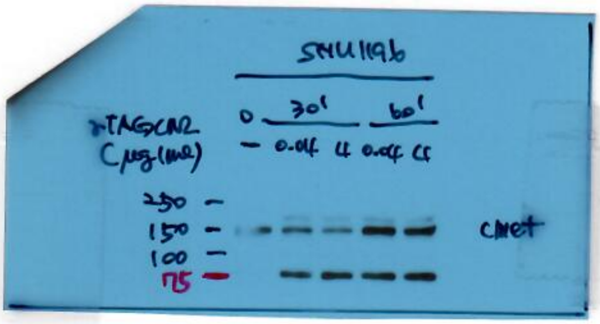
SNU119b
rTAGLN2 (μg/ml)
0 30' 60'
0.04 4 0.04 4
250
150
100
75
cmet

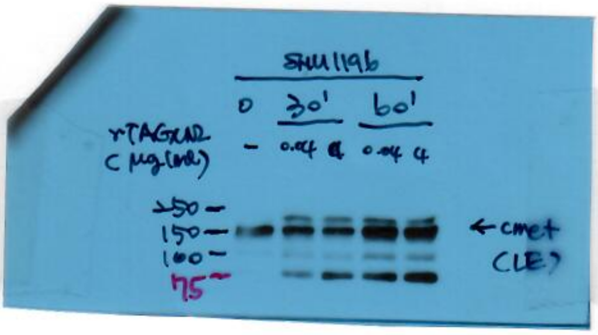
SNU119b
rTAGLN2 (μg/ml)
0 30' 60'
0.04 4 0.04 4
250
150
100
75
← cmet (LE)

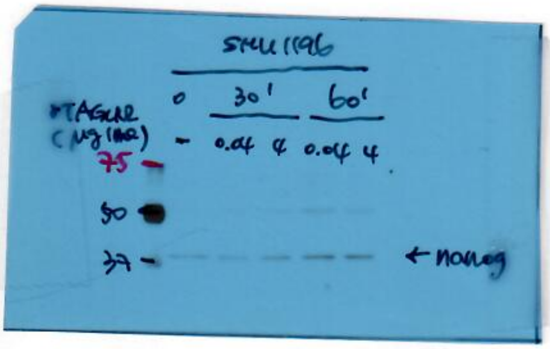
SNU119b
rTAGLN2 (μg/ml)
0 30' 60'
0.04 4 0.04 4
75
50
37
← nanog

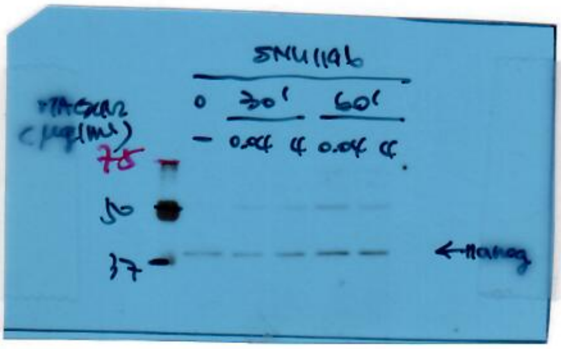
SNU119b
rTAGLN2 (μg/ml)
0 30' 60'
0.04 4 0.04 4
75
50
37
← nanog

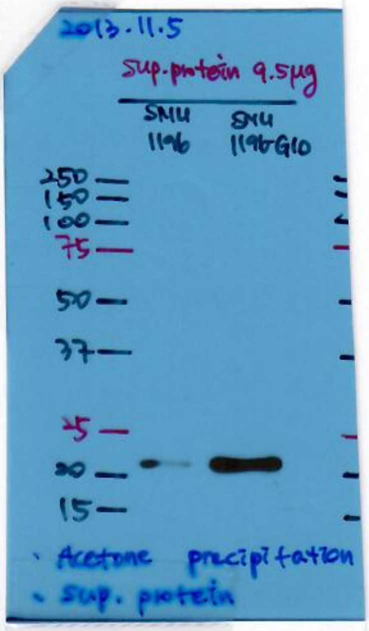

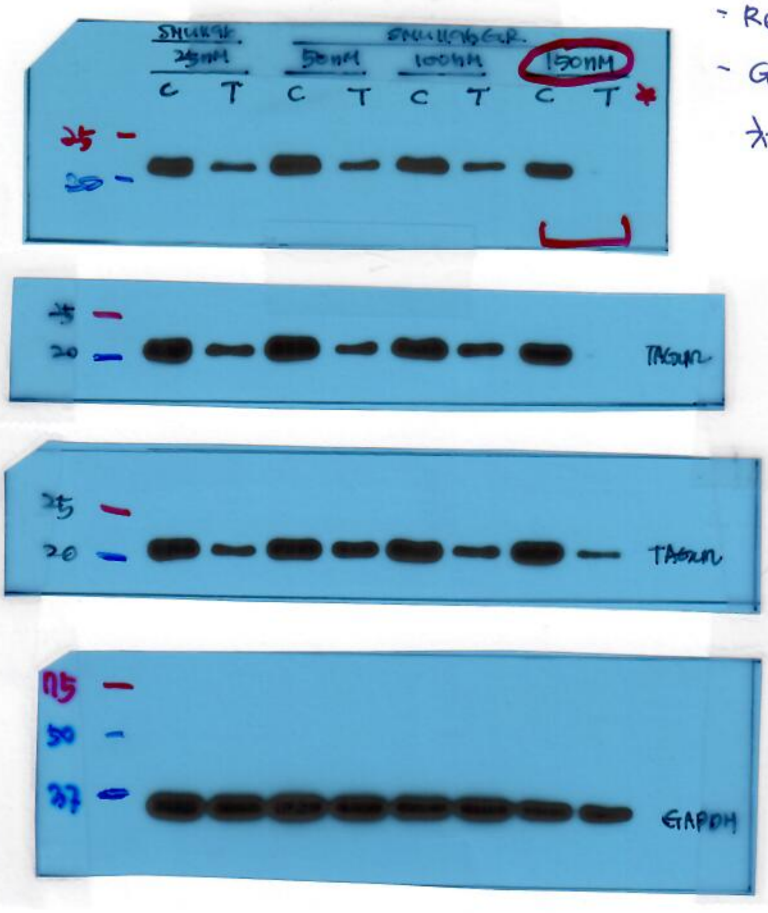

- C: control siRNA
- T: TAGLN2 siRNA.
- Reverse transfection 72h.
- GR cell에서 150nM siTAGLN2 처리시 확연히 inhibition 됨.

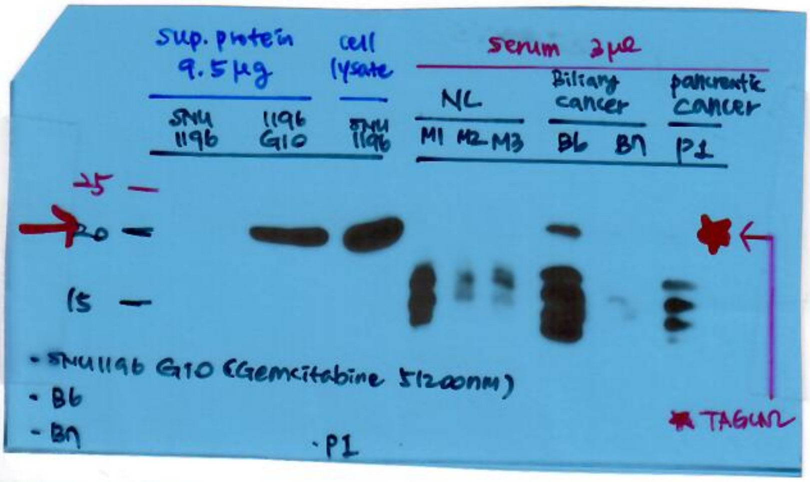
sup. protein
9.5 μg
cell lysate
serum 2μl
NL
Biliary cancer
pancreatic cancer
SNU 1196
1196 G10
SNU 1196
M1 M2 M3
B6 B7
P1
25
20
15
- SNU1196 G10 (Gemcitabine 51200nM)
- B6
- B7
- P1
TAGLN

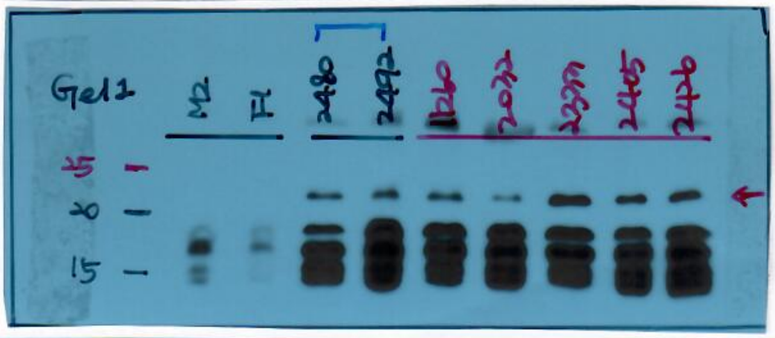
Gel1
M2
F1
25
20
15

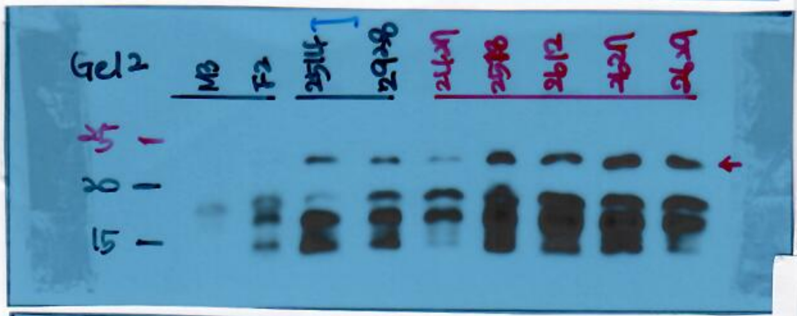
Gel2
M3
F2
25
20
15

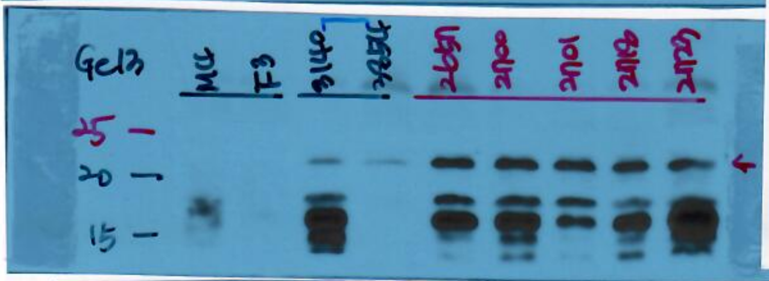
Gel3
M4
F3
25
20
15

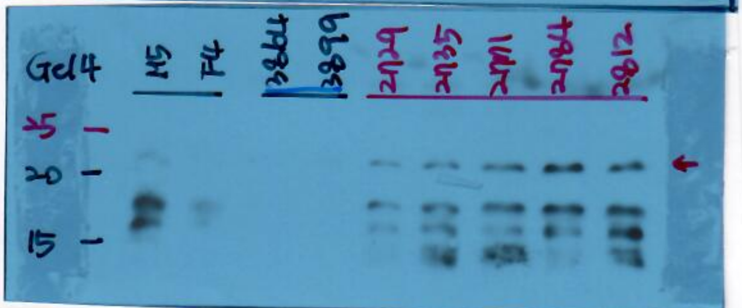
Gel4
M5
F4
25
20
15

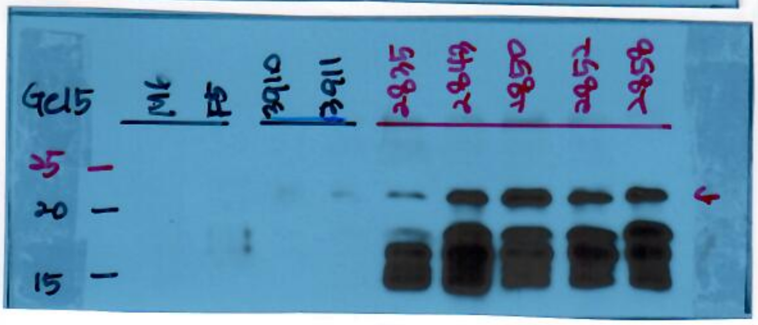
Gel5
M6
F5
25
20
15

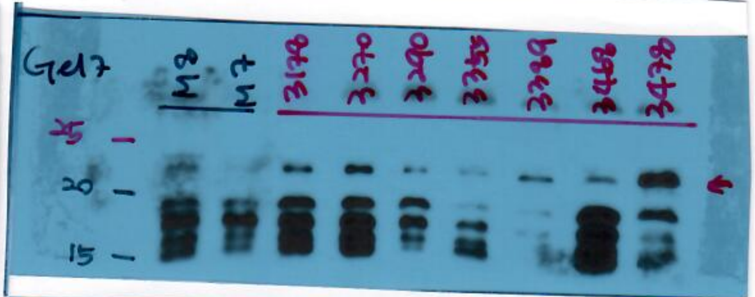
Gel7
M8
F7
25
20
15

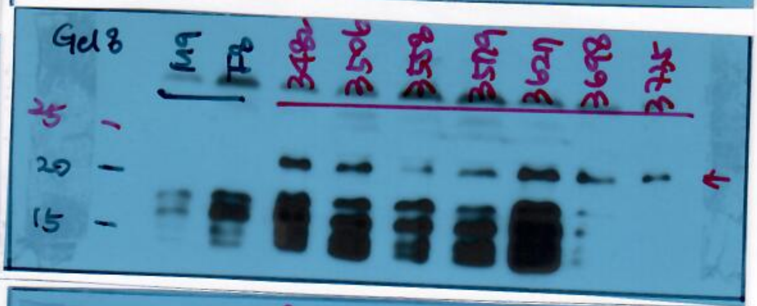
Gel8
M9
F8
25
20
15

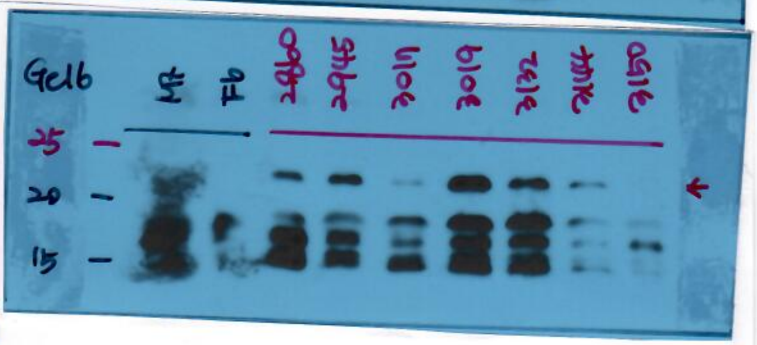
Gel6
M7
F6
25
20
15

진단마커로서의 TAGLN2 검증. (Serum)

① 건강 Normal 41 케이스 (Normal)

② 담도암 환자 48 케이스 (cancer)

- ✓ serum 3μl loading
- ✓ 12% SDS-PAGE
- ✓ 5% BSA blocking — 3hrs. RT
- ✓ Santacruz mouse TAGLN2 Ab. — 4°C. O/N.
- ✓ Blue : Normal
- ✓ Red : cancer

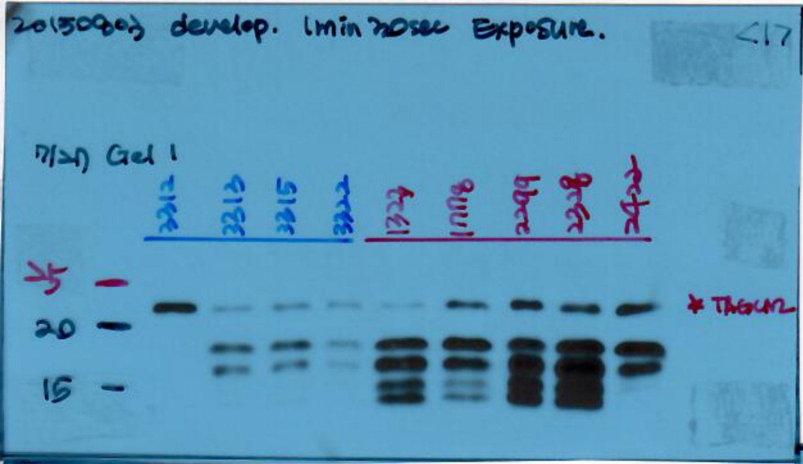

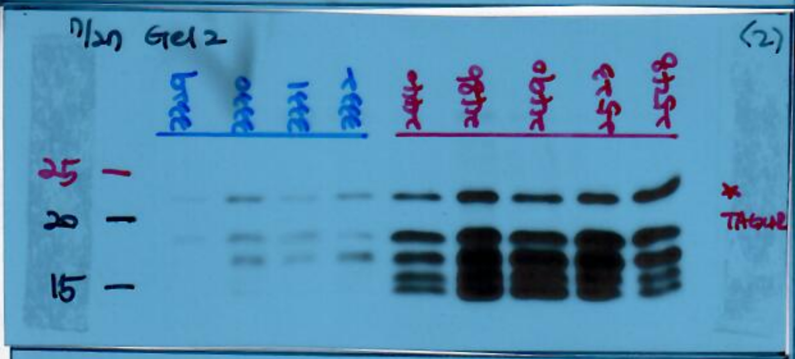

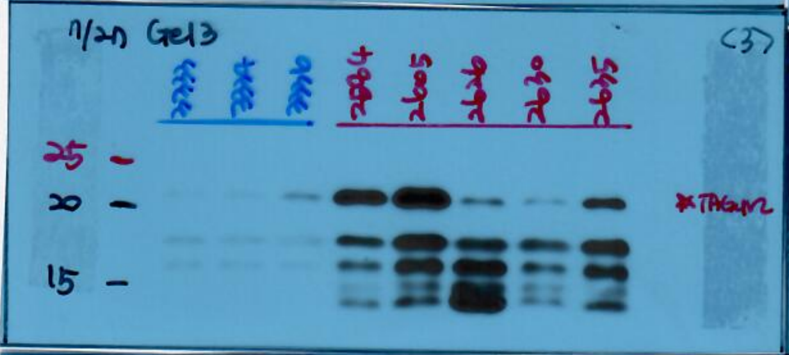

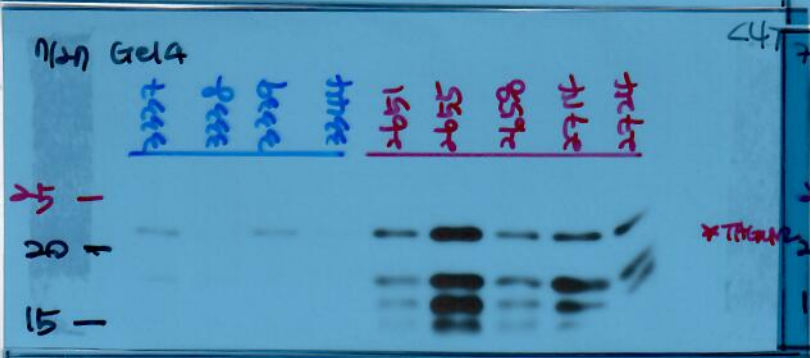

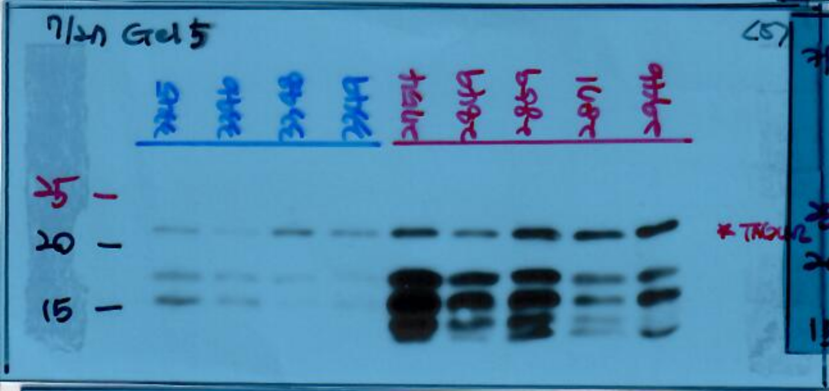

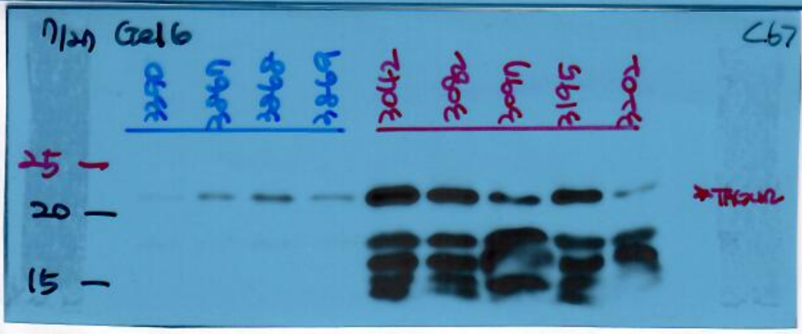

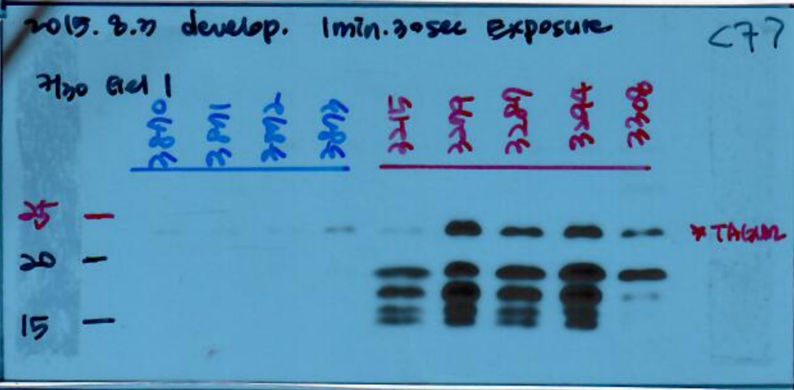

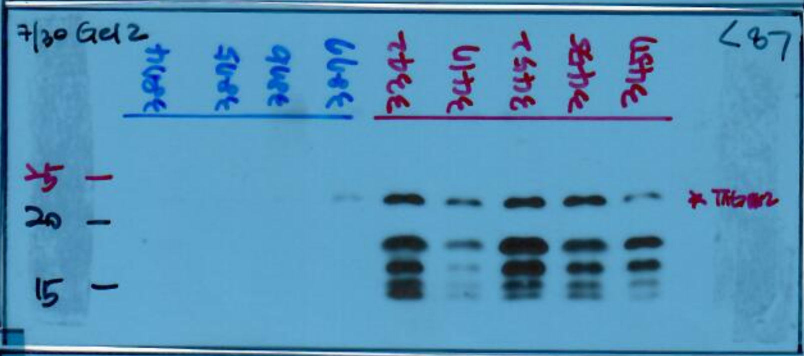

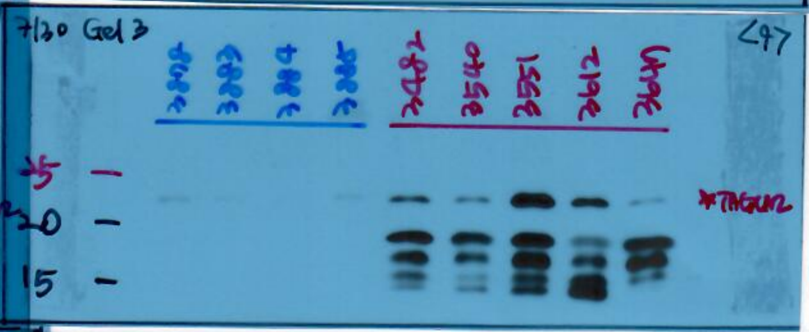

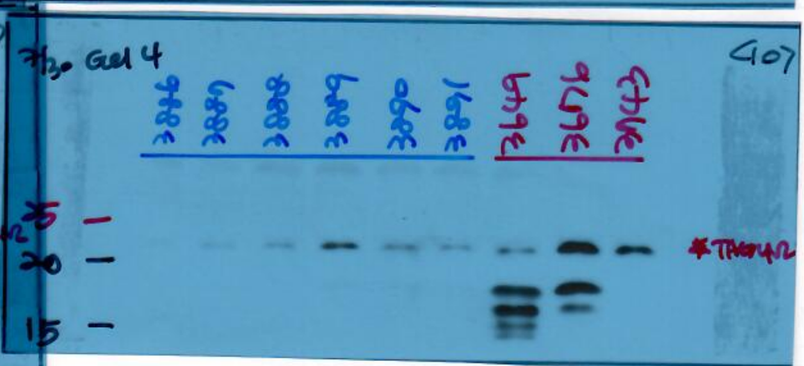

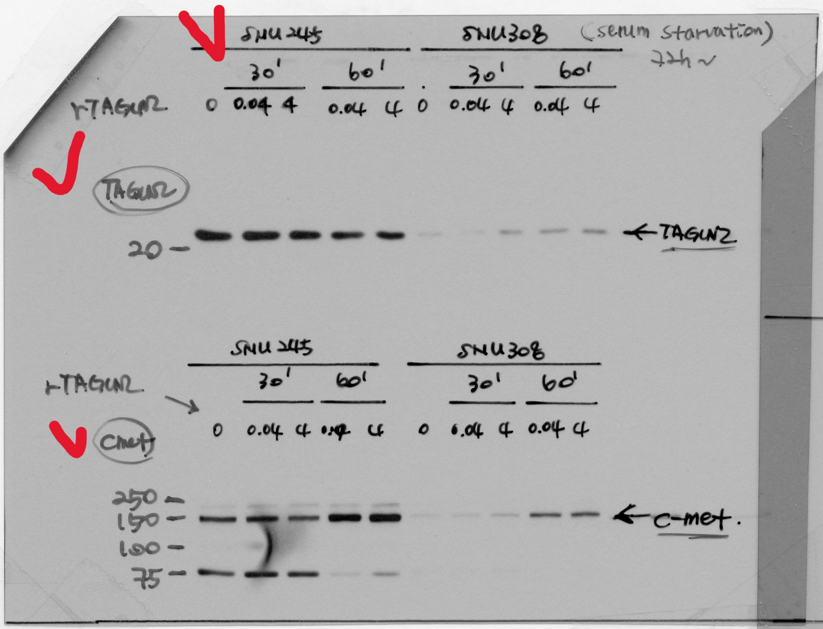
SNU245
SNU308
(serum starvation) 72h
30'
60'
30'
60'
rTAGLN2
0 0.04 4 0.04 4 0 0.04 4 0.04 4
TAGLN2
20 –
←TAGLN2
SNU245
SNU308
rTAGLN2
30'
60'
30'
60'
0 0.04 4 0.04 4 0 0.04 4 0.04 4
Cmet
250 –
150 –
100 –
75 –
← c-met.
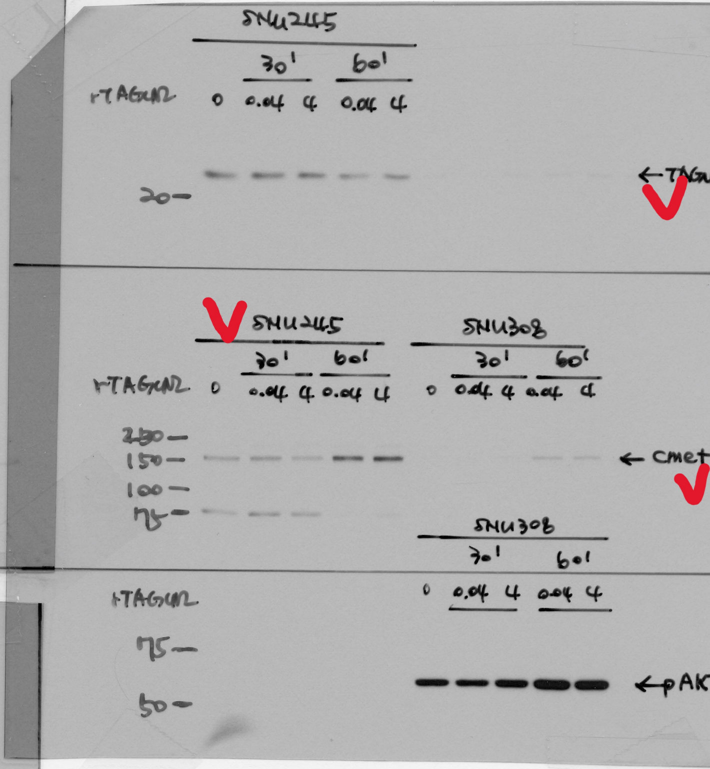
SNU245
30'
60'
rTAGLN2
0 0.04 4 0.04 4
20 –
SNU245
SNU308
30'
60'
30'
60'
rTAGLN2
0 0.04 4 0.04 4 0 0.04 4 0.04 4
250 –
150 –
100 –
75 –
← cmet
SNU308
30'
60'
rTAGLN2
0 0.04 4 0.04 4
75 –
50 –
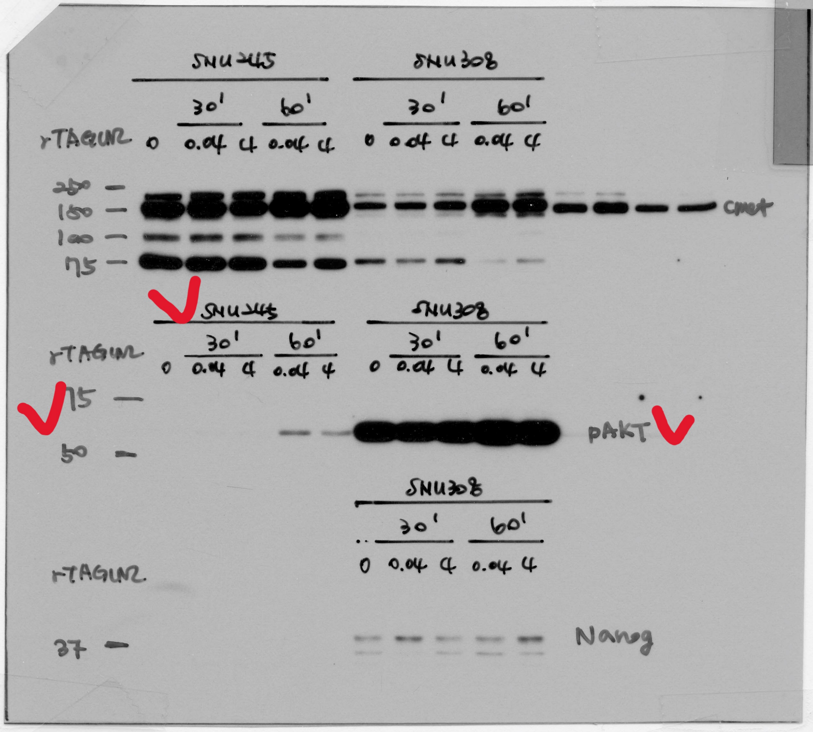
SNU245
SNU308
30'
60'
30'
60'
rTAGLN2
0 0.04 4 0.04 4 0 0.04 4 0.04 4
250 –
150 –
100 –
75 –
cmet
SNU245
SNU308
30'
60'
30'
60'
rTAGLN2
0 0.04 4 0.04 4 0 0.04 4 0.04 4
75 –
50 –
pAKT
SNU308
30'
60'
rTAGLN2
0 0.04 4 0.04 4
37 –
Nanog
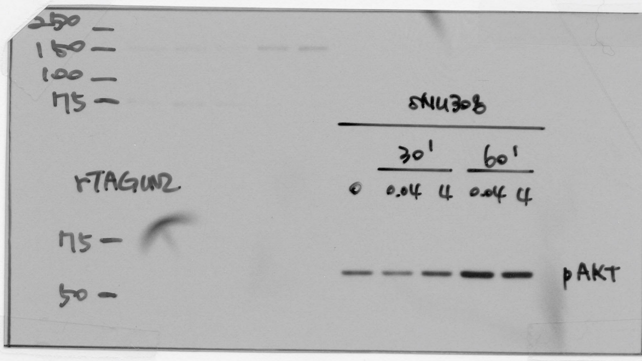
250 –
150 –
100 –
75 –
SNU308
30'
60'
rTAGLN2
0 0.04 4 0.04 4
75 –
50 –
pAKT
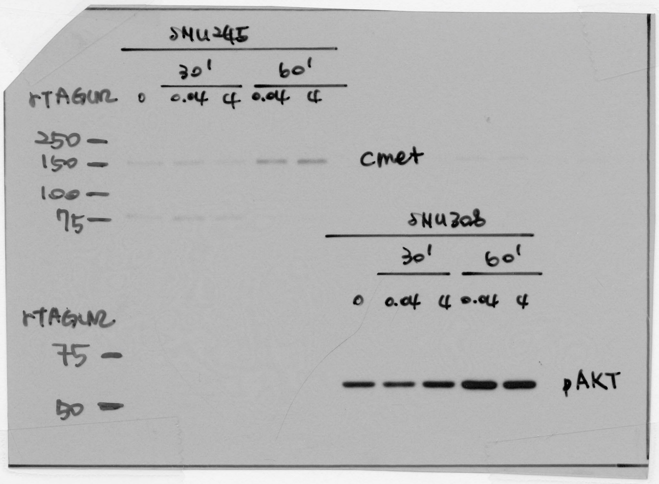
SNU245
30'
60'
rTAGLN2
0 0.04 4 0.04 4
250 –
150 –
100 –
75 –
cmet
SNU308
30'
60'
0 0.04 4 0.04 4
rTAGLN2
75 –
50 –
pAKT
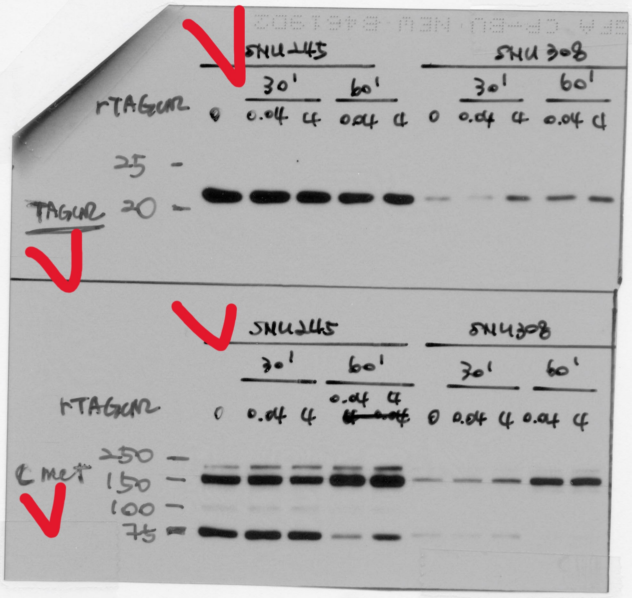
SNU245
SNU308
30'
60'
30'
60'
rTAGLN2
0 0.04 4 0.04 4 0 0.04 4 0.04 4
25 –
TAGLN2
20 –
SNU245
SNU308
30'
60'
30'
60'
rTAGLN2
0 0.04 4 0.04 4 0 0.04 4 0.04 4
Cmet
250 –
150 –
100 –
75 –

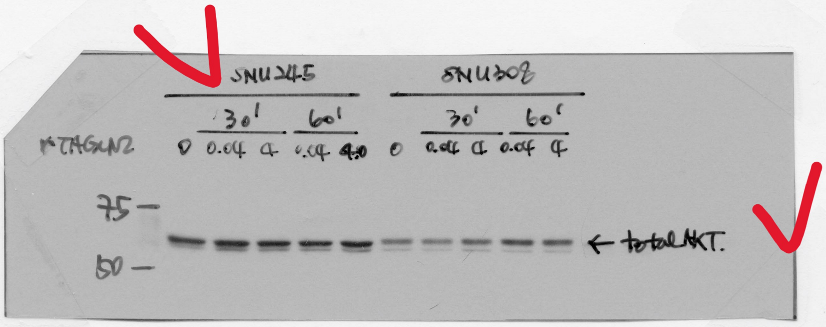
SNU245
SNU308
30'
60'
30'
60'
rTAGLN2
0 0.04 4 0.04 4.0 0 0.04 4 0.04 4
75
50
← total AKT.

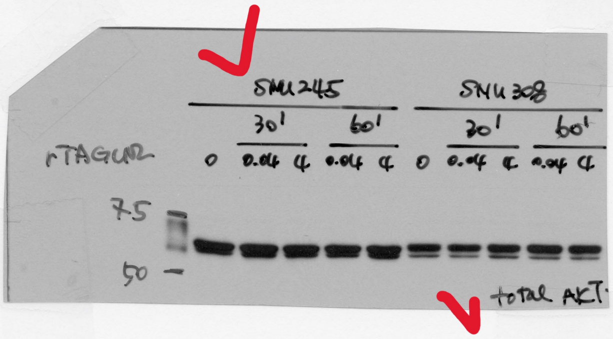
SNU245
SNU308
30'
60'
30'
60'
rTAGLN2
0 0.04 4 0.04 4 0 0.04 4 0.04 4
75
50
total AKT

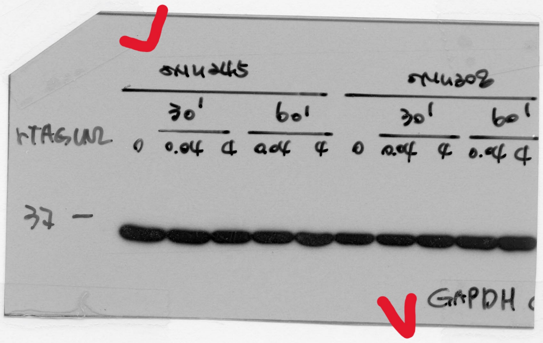
SNU245
SNU308
30'
60'
30'
60'
rTAGLN2
0 0.04 4 0.04 4 0 0.04 4 0.04 4
37
GAPDH

2023-11-22 SNU308. SNU869. - Adherent vs Sphere 1st revision 실험

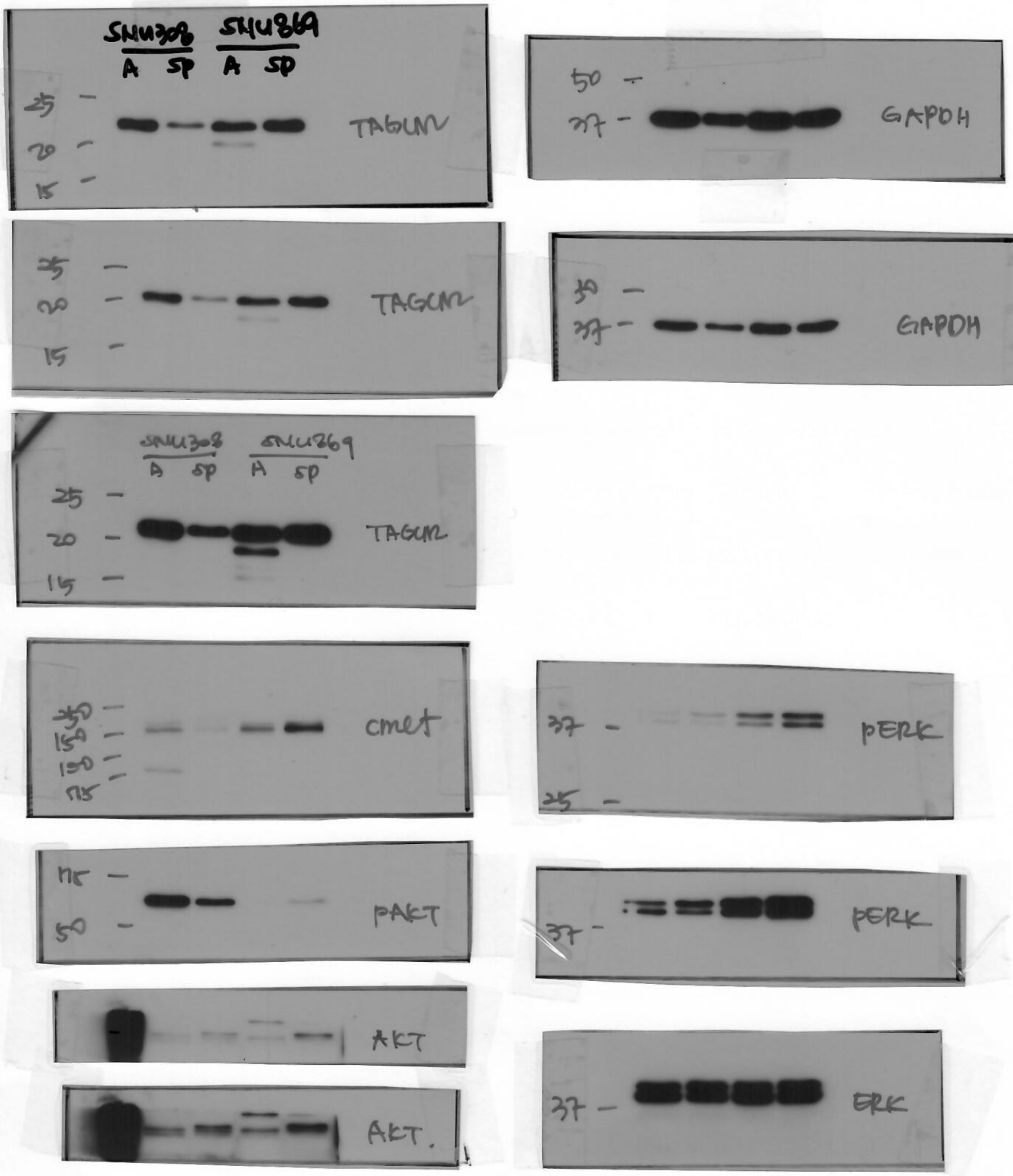

2024-01-17 SNU308 - SNU869 — Adherent vs sphere.

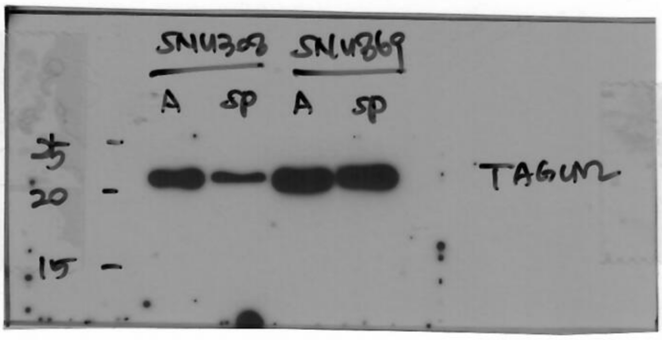

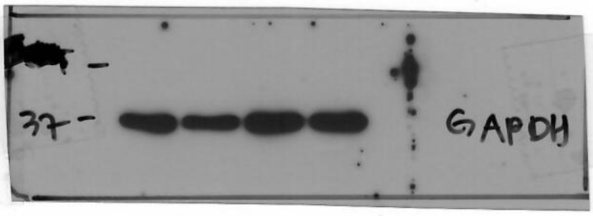

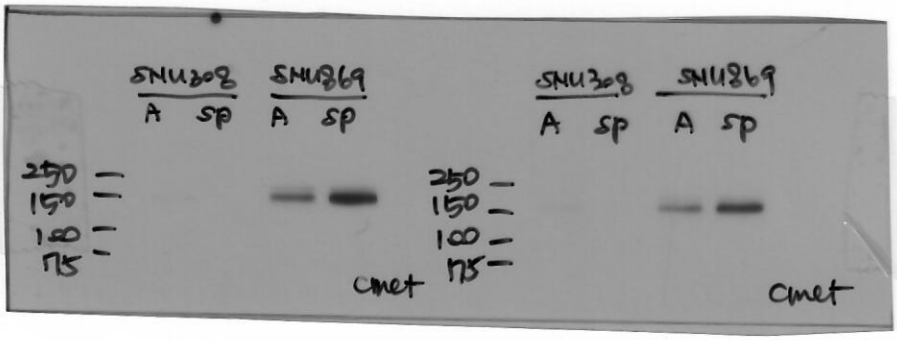

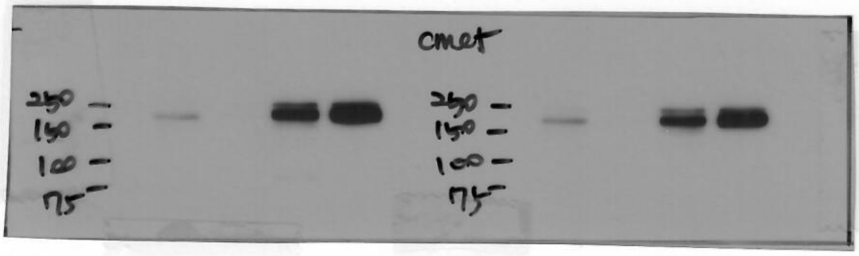

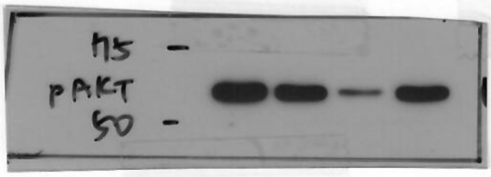

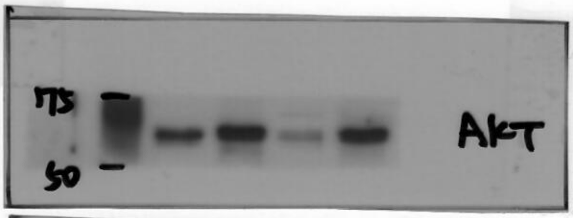

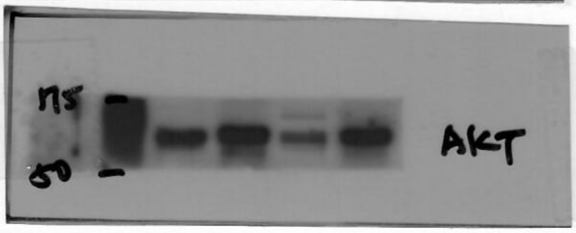

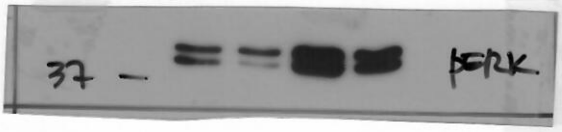

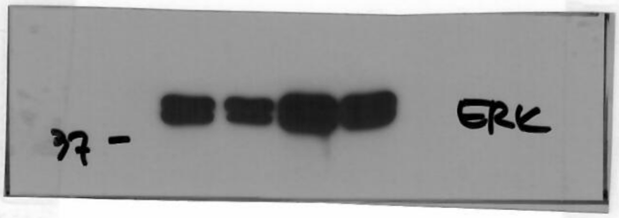

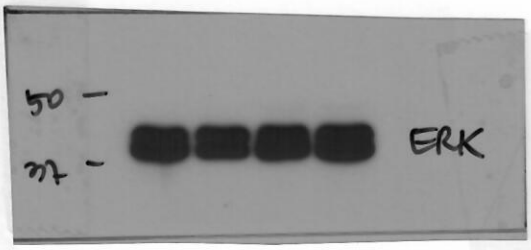

Supplement: Supplementary file 3 — Supplementary Material 3 [file 12885_2024_12082_MOESM3_ESM.pdf]
